# Biomechanical Investigation of Disturbed Hemodynamics-Induced Tissue Degeneration in Abdominal Aortic Aneurysms Using Computational and Experimental Techniques

**DOI:** 10.3389/fbioe.2019.00111

**Published:** 2019-05-31

**Authors:** Huseyin Enes Salman, Burcu Ramazanli, Mehmet Metin Yavuz, Huseyin Cagatay Yalcin

**Affiliations:** ^1^Biomedical Research Center, Qatar University, Doha, Qatar; ^2^Department of Mechanical Engineering, Middle East Technical University, Ankara, Turkey

**Keywords:** abdominal aortic aneurysm, rupture risk assessment, finite element analysis, computational fluid dynamics, fluid-structure interaction, particle image velocimetry, hemodynamics, experimental fluid mechanics

## Abstract

Abdominal aortic aneurysm (AAA) is the dilatation of the aorta beyond 50% of the normal vessel diameter. It is reported that 4–8% of men and 0.5–1% of women above 50 years of age bear an AAA and it accounts for ~15,000 deaths per year in the United States alone. If left untreated, AAA might gradually expand until rupture; the most catastrophic complication of the aneurysmal disease that is accompanied by a striking overall mortality of 80%. The precise mechanisms leading to AAA rupture remains unclear. Therefore, characterization of disturbed hemodynamics within AAAs will help to understand the mechanobiological development of the condition which will contribute to novel therapies for the condition. Due to geometrical complexities, it is challenging to directly quantify disturbed flows for AAAs clinically. Two other approaches for this investigation are computational modeling and experimental flow measurement. In computational modeling, the problem is first defined mathematically, and the solution is approximated with numerical techniques to get characteristics of flow. In experimental flow measurement, once the setup providing physiological flow pattern in a phantom geometry is constructed, velocity measurement system such as particle image velocimetry (PIV) enables characterization of the flow. We witness increasing number of applications of these complimentary approaches for AAA investigations in recent years. In this paper, we outline the details of computational modeling procedures and experimental settings and summarize important findings from recent studies, which will help researchers for AAA investigations and rupture mechanics.

## Introduction

Abdominal aortic aneurysm (AAA) is the dilatation of the abdominal aorta beyond 50% of the normal vessel diameter, due to degeneration of the arterial wall (McGloughlin Timothy and Doyle Barry, 2010). It is reported that 4–8% of men and 0.5–1% of women above 50 years of age bear an AAA and it accounts for ~15,000 deaths per year in the United States alone (Sakalihasan et al., 2005; Kontopodis et al., 2014). AAA may result in rupture of the vessel wall, which is a fatal surgical emergency because of reduced blood flow to vital organs and hematocele (i.e., swelling caused by blood collecting in a body cavity). AAA rupture is the most catastrophic complication of the aneurysmal disease that is accompanied by a striking overall mortality of 80% (Bengtsson and Bergqvist, 1993). Unfortunately, clinical symptoms for impending AAA rupture are not observed for nearly 75% of the patients (Anjum et al., 2012).

Current clinical practice is surgical repair if the diameter of the AAA is >5.5 cm or the growth rate is over 1 cm/year and to follow up AAAs with diameters < 5.5 cm at 6-month intervals (Cosford and Leng, 2007). It is reported that only 25% of AAAs ruptured in the patient's lifetime (Darling et al., 1977), and 13% of AAAs with a diameter of < 5 cm are ruptured. For this reason, AAA diameter is not the sole indicator for the rupture and other factors should also be considered for the rupture risk assessment, together with the size of AAA.

The precise mechanisms leading to AAA rupture remains unclear. From a purely mechanical point of view, rupture of an AAA occurs when the mechanical stresses (i.e., internal forces per unit area) acting on the aneurysm exceeds the ability of the wall tissue to withstand these stresses (i.e., the wall's failure strength). Wall shear stress (i.e., WSS, the frictional force exerted by blood flow on the luminal surface) is also thought to play an important role (Peattie et al., 1996). While the blood flow in normal aorta (diameter within 2–2.5 cm) is mainly anterograde with high WSS, during AAA, circulatory flows emerge within the vessel. Hence, flow becomes disturbed with oscillatory characteristics leading to low WSS (Tanweer et al., 2014). The disturbance of flow in AAA is believed to contribute to the progression of the disease by activating the inflammatory markers of the endothelial cells lining the vessel wall which might lead to degeneration and weakening of the vessel wall (Franck et al., 2013). Therefore, prediction of the growth course of an AAA and precise determination of the rupture risk is a very challenging problem and attracts substantial attention.

Currently, there is no established conclusive approach for AAA rupture risk assessment, rather than the limited clinical guidelines for the AAA size. Ideally, the problem needs to be investigated from a biomechanical point of view, for a comprehensive wall stress, wall strength, and hemodynamic analyses. Computational modeling based on finite element analysis (FEA) is a powerful technique for estimating mechanical behavior of materials (i.e., mechanical behavior is the amount of stresses and deformations in a substance under external forces). This is especially beneficial in medical research where in most cases direct measurement of *in-vivo* mechanical response of diseased tissue is not possible. In this technique, first an accurate representation of the problem geometry is generated. The second step is defining the conditions in the model boundaries. The third step is defining different zones in the geometry, and assigning appropriate material properties for each zone and the fourth step is dividing zones into smaller elements (meshing). The last step is numerically solving appropriate governing equations in each small element to obtain a solution field of interest in the entire geometry. Each of these steps are critically important for solution accuracy.

Computational modeling has been used frequently in recent years to investigate rupture mechanics for AAA tissue. Comparison of ruptured and electively repaired AAA cases via FEA, revealed elevated wall stress levels for ruptured tissue (Fillinger et al., 2002). Several observations on excised tissue suggest that AAA formation is accompanied by an increase in wall stress as well as a corresponding decrease in wall strength (Vorp et al., 1996; Raghavan et al., 2000) and rupture point on AAA is usually coincides with peak wall stress locations (Fillinger et al., 2002). In very few studies where previous medical images for ruptured AAA tissue were available, patient-specific FEA could successfully identify known exact future rupture locations as high wall stress regions (Doyle et al., 2014). The FEA results prove the potential significant contribution of computational analysis for rupture risk assessment for AAA.

As computational methods have widely been used in investigation of hemodynamics and mechanical behavior of arterial tissue, the experimental techniques are also utilized in characterization of flow dynamics through AAAs. Both approaches are crucial and complement each other with offering in depth analysis where the level of its intensity and accuracy depend on the assumptions in computational models and simplifications in experimental methods. In line with the aforementioned computational studies, many experimental investigations of the hemodynamics through AAAs have been conducted in literature, and various qualitative and quantitative flow measurement techniques have been utilized for that purpose. A typical experimental set up for the analysis of hemodynamics contains physiological flow circulatory system including pump, piping, and pressure compliance, test section that contains artery model (phantoms), blood mimicking fluid, and flow measurement systems (like particle image velocimetry) to track the movement of fluid particles to calculate the velocities first, and then WSS levels on the phantoms. Previously, this approach has been adapted by several researchers to investigate AAA rupture mechanism (Tanweer et al., 2014; Wang et al., 2016).

In this paper, we will summarize the techniques utilized in experimental and computational studies of AAAs. We will briefly explain important aspects of the modeling procedure and how to set up and run relevant simulations and also, how to generate a flow circulation set up with particle image velocimetry (PIV). In addition, we will summarize important findings for experimental and computational biomechanical assessments of AAAs.

## Computational Investigation of Biomechanics of AAAs

Computational models enable researchers to approximate biomechanical behavior of tissue stress and blood flow hemodynamics under realistic conditions. The models are generated by defining the conditions on the boundaries and numerically solving governing equations in fluid and solid domains. Generation of the problem geometry, setting up solid and fluid models, coupling the solutions through FSI procedure are important steps which contribute to enhance the reliability and accuracy of the biomechanical assessment as will be mentioned in the forthcoming sections.

### Mathematical Details of the Problem

#### Geometry of the Problem

**Figure 1:**
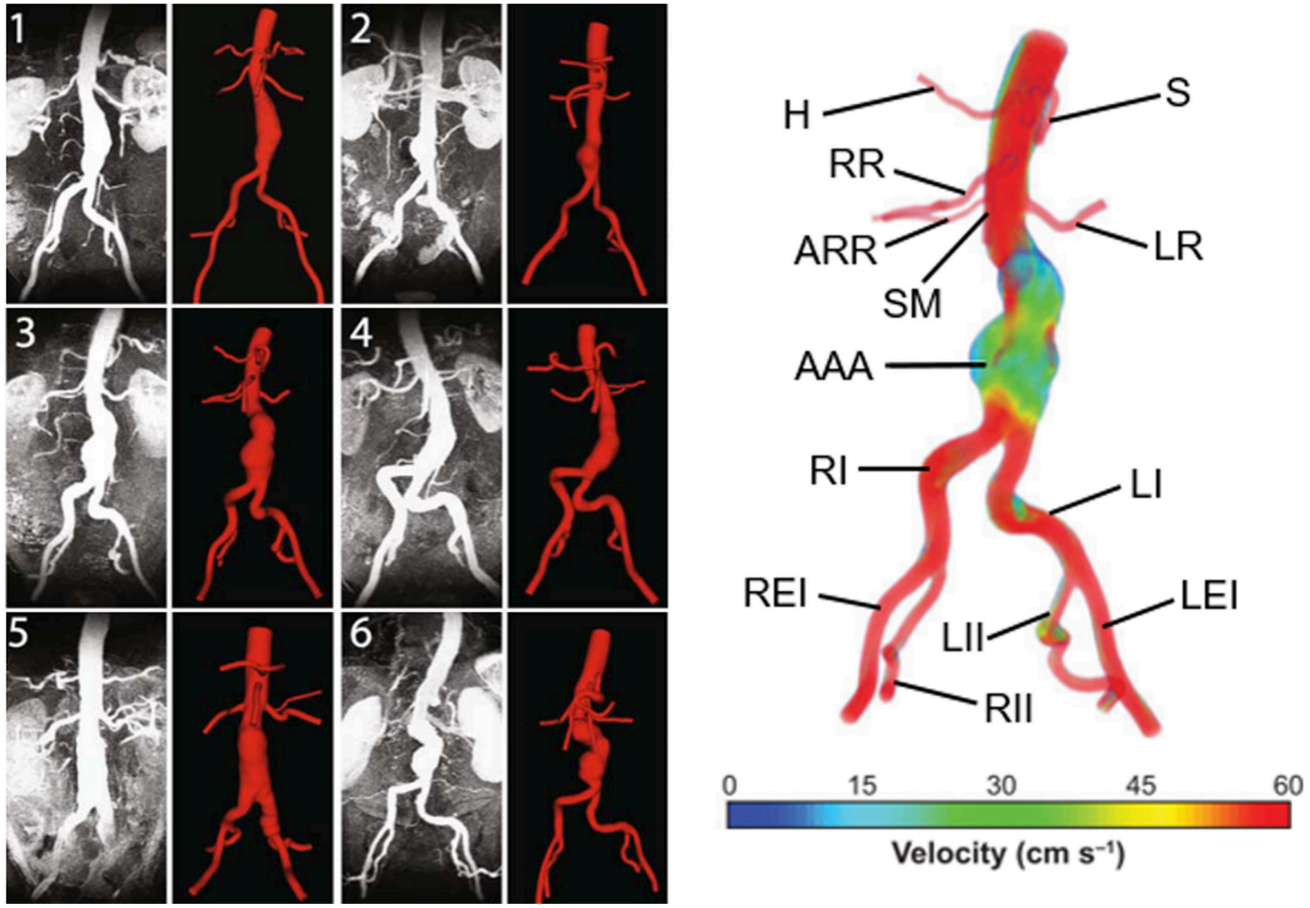
Six different patient-specific medical images and corresponding reconstructed AAA geometries. The branching arteries and disturbed AAA flow in the 3rd patient-specific medical image are described at the right side. Compared to undilated vessel, the flow hemodynamics in the AAA sac changed significantly. The artery labels are as follows: H, Hepatic artery; S, Splenic artery; RR, Right renal artery; ARR, Accessory right renal artery; SM, Superior mesenteric artery; LR, Left renal artery; AAA, Abdominal aortic aneurysm; RI, Right iliac artery; LI, Left iliac artery; REI, Right external iliac artery; RII, Right internal iliac artery; LII, Left internal iliac artery; LEI, Left external iliac artery [The figure is adapted from Les et al. (2010) and used with permission].

AAA has unique, patient-specific and complex geometry with a wall thickness around 1.5 mm (Raghavan et al., 2004) and mostly does not represent an axisymmetric form. Healthy abdominal aortic diameter is around 2 cm and in case of dilatation, AAA diameter can expand up to 9 cm. Proximal AAA neck angle, distal iliac bifurcation angle, heterogeneous wall thickness are patient-specific geometric parameters. Left and right renal arteries, left and right iliac arteries and superior mesenteric artery are the main branching arteries in abdominal aorta. In Figure 1, different patient-specific AAA geometries, branching arteries and disturbed flow in AAA sac are presented.

**Figure 2:**
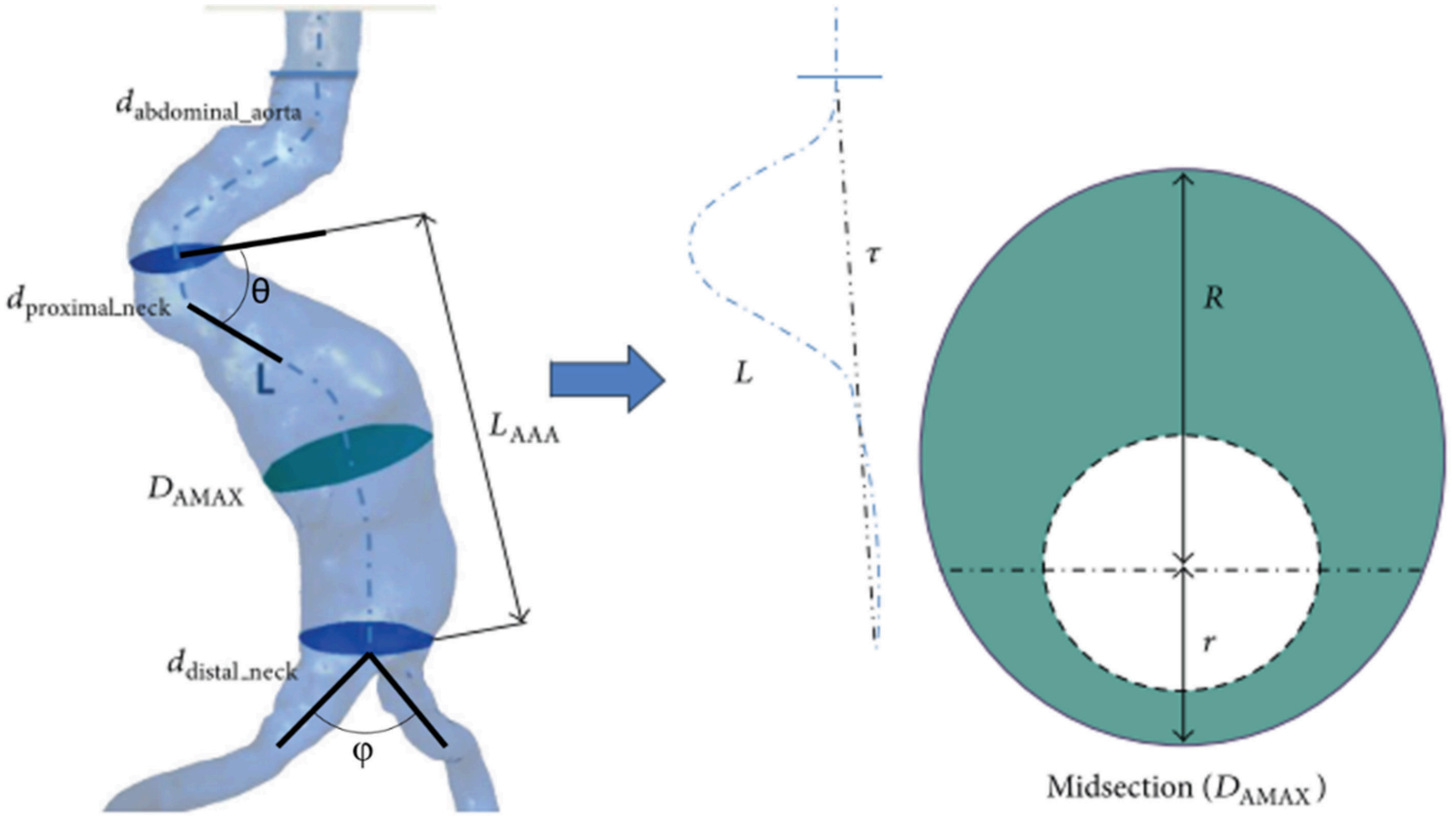
Main geometric parameters for a patient-specific AAA geometry. *L*_AAA_, Aneurysm length; *D*_AMAX_, Maximum aneurysm diameter; *d*_proximal_neck_, Inlet diameter of AAA sac; *d*_distal_neck_, Outlet diameter of AAA sac; *d*_abdominal_aorta_, Normal abdominal aorta diameter; θ, Proximal neck angle; ϕ, Iliac bifurcation angle; *L*, Absolute length of tortuous vessel; τ, Imaginary straight line starting at the center of normal abdominal aorta and ending at the iliac bifurcation. The midsection at the location of the maximum AAA diameter (*D*_AMAX_) is represented at the right side. At the maximum diameter midsection, *r* and *R* are the radii measured from center of the undilated portion (i.e., normal abdominal aorta) to the posterior and anterior walls, respectively [The figure is adapted from Soudah et al. (2013) and used with permission].

Main geometric parameters for a patient-specific AAA are presented in Figure 2. The AAA asymmetry is defined using the relation defined by Vorp et al. (1998). β is the parameter of asymmetry defined according to the central axis of undilated diameter, as shown in Figure 2. In Equation (1), *r* and *R* are the radii measured from center of the undilated portion to the posterior and anterior walls, respectively.

(1) $$\begin{array}{l} \beta =\frac{r}{R} \end{array}$$

In addition to the maximum aneurysm diameter, tortuosity, curvature, proximal neck angle, iliac bifurcation angle, and complex shape of the aneurysm itself are influencing factors on the wall stress (Stringfellow et al., 1987; Vorp et al., 1998; Hua and Mower, 2001). Up to 80% of AAA rupture are observed on the posterior wall (Darling et al., 1977), demonstrating the importance of asymmetry and curved AAA geometry in rupture. Loss of curvature on the posterior wall and AAA asymmetry are suggested as dominant wall stress increasing factors (Scotti et al., 2008).

#### Generation of Model Geometry

Magnetic resonance imaging (MRI), three-dimensional (3D) ultrasound, computerized tomography (CT) are the most common medical imaging techniques used to extract the realistic geometry of AAAs. At present, primary imaging method is CT. However, due to its high cost, significant radiation dose, injection of ionated contrast medium associated with nephrotoxity and relatively less availability, alternative methods such as 3D ultrasound can be used to widespread the biomechanical assessments.

**Figure 3:**
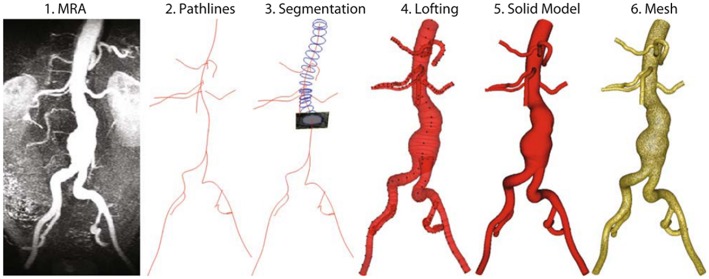
Reconstruction procedure for a patient-specific AAA model [The figure is adapted from Les et al. (2010) and used with permission].

After obtaining patient-specific medical images in DICOM (Digital Imaging and Communications in Medicine) format, 3D AAA models can be reconstructed using segmentation software such as MIMICS (Materialize, Leuven, Belgium), VESSEG (Carniege Mellon University, Pittsburgh, PA), ImFusion Suite (ImFusion GmbH, Munich, Germany), and open-source software such as SimVascular, VMTK, and ITK-SNAP. Lumen and AAA wall are segmented separately to differentiate these zones. Segmented 3D model might include protrusions and tight internal corners, which requires application of smoothing algorithms, to prepare a suitable geometry for FEA simulations. Autodesk MeshMixer, AngioLab, and MeshLab softwares can be used for further smoothing and mesh optimization processes. For smoothing process, different algorithms can be applied such as Laplacian (Field, 1988), HC Laplacian (Vollmer et al., 2001), and Taubin's low pass filter (Taubin, 1995). In Figure 3, the reconstruction procedure of a patient-specific AAA model is given.

#### Governing Equations in Solid Domain

The governing equation in solid domain is the momentum conservation given in Equation (2) (Scotti et al., 2008).

(2) $$\begin{array}{l} \nabla ▪\tau_{s}+{\text{f}}_{s}^{B}=\rho_{s}\ddot{{\text{d}}_{s}} \end{array}$$

In Equation (2), **τ**_*s*_ is solid stress tensor; ${\text{f}}_{s}^{B}$ is body forces term per unit volume; ρ_*s*_ is AAA wall mass density; and $\ddot{{\text{d}}_{s}}$ is local acceleration of solid. Lagrangian description is typically used to track the deformation of the solid domain (Donea et al., 1982; Bathe and Zhang, 2004). Neglecting the gravitational forces (${\text{f}}_{s}^{B}$) is a commonly used approach due to their insignificant effect on wall stresses (Wolters et al., 2005).

#### Important Stress Parameters for Solid Domain

For the solid domain, principal wall stresses (σ_1_, σ_2_, σ_3_), Von Mises stress and wall displacements are critical parameters for AAA rupture risk assessment. The peak wall stresses are better indicators when compared to the maximum AAA diameter, since rupture is the mechanical failure of the wall where the strength of wall is not sufficient to withstand the peak wall stress (Fillinger et al., 2003; Wolters et al., 2005). Von Mises stress is a measure used for failure prediction based on three principal stresses as given in Equation (3).

(3) $$\begin{array}{l} \frac{1}{2}\left[{\left(\sigma_{1}-\sigma_{2}\right)}^{2}+{\left(\sigma_{2}-\sigma_{3}\right)}^{2}+{\left(\sigma_{3}-\sigma_{1}\right)}^{2}\right]>{\sigma_{y}}^{2} \end{array}$$

In Equation (3), the term in the left side is square of Von Mises stress, σ_*i*_ is the local principal stress and σ_*y*_ is the uniaxial failure strength of the wall (Scotti et al., 2008).

#### Incorporation of Intraluminal Thrombus (ILT) to the Models

In solid domain, the presence of intraluminal thrombus (ILT) is an influencing factor for the wall stress depending on its shape, size, and material properties (Mower et al., 1997; Di Martino et al., 1998; Di Martino and Vorp, 2003). Flow hemodynamics is also closely related with the ILT formation on AAA wall. ILT contains immune and inflammatory response agents affecting the evolution of the disease (Adolph et al., 1997). In a related clinical study, ILT is observed near the site of rupture for 80% of the autopsies (Simão da Silva et al., 2000), as being a source of proteolytic activity, local wall thinning, wall weakening, and hypoxia (Swedenborg and Eriksson, 2006; Houard et al., 2007). ILT morphology can exhibit a layered structural material behavior or fluid-like homogeneous medium. It becomes a poroelastic material at a certain state of maturity which is affecting blood transport (Tong and Holzapfel, 2015). Flow stagnation and associated low WSS, high residence time of platelets and monocytes in AAA sac contribute to high potential of wall-cell adhesion forming ILT (Kelsey et al., 2016). In a recent study, Di Achille et al. (2016) predicted ILT formation and progression sites in patient-specific AAA models, using a phenomenological metric of thrombus deposition potential which is indicating a balance between the thrombogenicity and hemodynamic shear forces on the wall. The relative overlap between predicted and actual thrombus covered areas in six patient-specific AAA models was reported around 80 ± 16% (Di Achille et al., 2016). Therefore, ILT formations on vessel walls should be assigned with appropriate material constants with accurate geometric representations for model accuracy since ILT significantly affects both the wall mechanics and flow hemodynamics, indicating the importance of interactions between solid and fluid domains.

#### Wall Material Properties

Aorta consists of three layers: the intima, media, and adventitia (Lasheras, 2006). Intima and adventitia are the inner and outer layers, respectively. The distribution of layer thicknesses has a ratio about 20:47:33 for intima: media: adventitia (Humphrey and Holzapfel, 2012). Elastic modulus ratio for intima: media: adventitia is ~1:3:2 (Khanafer and Berguer, 2009; Gao et al., 2013). The highest stresses are observed in the media layer due to the normal pressure of cyclic flow (Simsek and Kwon, 2015). The maximum wall deformation can reach up to 2.2 mm at the systolic phase (Canchi et al., 2018). One of the possible reasons of AAA formation is reported as the medial loss in the arterial wall with degeneration of smooth muscle cells (Humphrey and Taylor, 2008). In case of AAA wall weakening depending on the media loss, elastic modulus of the media layer can be reduced by 20 times (Feng et al., 2008). Modeling AAA wall as a single-layered structure is a commonly used approach for simplification of the problem.

#### Governing Equations in Fluid Domain

Flow velocity, pressure and wall shear stress (WSS) exerted by fluid viscous forces are determined by solving the Navier-Stokes and continuity equations. For an incompressible and homogeneous fluid, the Navier-Stokes equations can be defined using the Arbitrary Lagrangian Eulerian (ALE) description given in Equation (4) and continuity equation given in Equation (5) (Donea et al., 1982; Zhang et al., 2003; Scotti and Finol, 2007).

(4) $$\begin{array}{lll} \rho_{f}\frac{\partial \text{v}}{\partial t} & + & \rho_{f}\left(\text{v}-\text{w}\right)▪\nabla \text{v}-\nabla ▪\tau_{f}={\text{f}}_{f}^{B} \end{array}$$

(5) $$\begin{array}{ll} \nabla & ▪\text{v}=0 \end{array}$$

The fluid velocity vector is denoted by **v**; time is denoted by *t*; the velocity of the fluid domain (i.e., moving coordinate velocity primarily due to FSI) is denoted by **w**; fluid stress tensor is denoted by **τ**_*f*_; and body forces term is denoted by ${\text{f}}_{f}^{B}$. The fluid stress tensor (**τ**_*f*_) is defined in Equation (6), in terms of fluid pressure (*p*), Kronecker delta (δ_*ij*_), dynamic viscosity (μ), and strain rate (ε_*ij*_). The strain rate can be written in terms of velocity vector (**v**), as given in Equation (7). The effect of gravitational acceleration is not critical and the body forces on the fluid (${\text{f}}_{f}^{B}$) can be neglected (Scotti et al., 2008).

(6) $$\begin{array}{l} \tau_{f}=-p\delta_{ij}+2\mu \varepsilon_{ij} \end{array}$$

(7) $$\begin{array}{l} \varepsilon_{ij}=\frac{1}{2}\left(\nabla \text{v}+{\nabla \text{v}}^{T}\right) \end{array}$$

#### Important Hemodynamic Parameters for Fluid Domain

In fluid domain, wall shear stress (WSS), oscillatory shear index (OSI), intraluminal pressure, flow path, and flow velocity are of main interest. Due to complex AAA geometries, WSS has spatially and temporally complicated distributions (Arzani and Shadden, 2015). WSS is a measure of flow-driven tangential forces per unit area on the AAA wall. The magnitude of WSS at the fluid-structure interface can be determined by multiplying the viscosity (μ) with the local shear rate ($\dot{\gamma }$) (Wolters et al., 2005). In large arteries, WSS amplitudes typically range from 1 to 5 Pa, therefore WSS values smaller than 1 Pa are evaluated as low WSS (Ene-Iordache and Remuzzi, 2012; Qiu et al., 2018). Time averaged WSS (TAWSS) can be determined as given in Equation (8) where *T* is the integration period (Arzani et al., 2014; Arzani, 2018).

(8) $$\begin{array}{l} TAWSS=\frac{1}{T}\int_{t-T}^{t}|WSS|dt \end{array}$$

OSI is a measure defining the unidirectionality of shear stress which is sensitive to turbulence and it can be determined as given in Equation (9) (Arzani et al., 2017).

(9) $$\begin{array}{l} OSI=\frac{1}{2}\left(1-\frac{\left|\frac{1}{T}\int_{t-T}^{t}WSSdt\right|}{\frac{1}{T}\int_{t-T}^{t}|WSS|dt}\right) \end{array}$$

When OSI is zero, it is indicating that shear stress is unidirectional. If OSI is 0.5, it means that time average of shear stress is zero. Endothelial cell activation potential (ECAP) is the ratio of OSI and TAWSS as given in Equation (10). ECAP is used to characterize ILT susceptibility on AAA wall (Di Achille et al., 2014). Critical threshold value of ECAP is stated as 1.4 Pa^−1^, where above this value, there is a high potential of ILT formation (Kelsey et al., 2016). Therefore, as ECAP increases due to emergence of circulatory flows in diseased states, there is a higher chance of wall inflammation.

(10) $$\begin{array}{l} ECAP=\frac{OSI}{TAWSS} \end{array}$$

#### Fluid Properties and Flow Regime

Blood has non-Newtonian characteristics where the viscosity decreases with increased shear rate. AAA flow is inherently pulsatile and has a turbulent nature particularly at mid-diastolic phase. At the systolic phase, peak blood flow velocity in AAA can reach up to 20 cm/s (Casciaro et al., 2018). Larger aneurysmal diameters lead to higher turbulence intensity and large recirculating vortices increasing the blood residence time in the aneurysm sac, particularly at the diastolic phase of the physiological pulsatile flow (Khanafer et al., 2007).

In many studies, AAA flow is considered as unsteady laminar flow (Scotti et al., 2008; Chandra et al., 2013; Morris et al., 2013; Soudah et al., 2013; Owen et al., 2016) due to not exceeding the threshold Reynolds number (2000–2300) for transition to turbulence in pipe flow. However, complex flow geometry, sudden lumen expansion in AAA sac and pulsatile nature of flow are the factors that might trigger the turbulence in low Reynolds numbers (Poelma et al., 2015). In order to resolve the turbulent effects accurately, Les et al. (2010) and Arzani et al. (2014) applied more demanding direct numerical simulation (DNS) approach, and Khanafer et al. (2007) used *k*-ω turbulence model.

#### Biochemical Transport

Oxygen (Sun et al., 2009), low density lipoproteins (Choudhury et al., 2019), and chemical species transported by blood flow have an influence on the AAA progression and ILT formation on the wall. Shear rate is an Eulerian measure that cannot quantify particle transport (Shadden and Arzani, 2015). Locally high surface concentration of chemical species are not always corresponds to the regions with low WSS that are determined using Eulerian mass transport (Choudhury et al., 2019). The residence time and flow path of the micro-particles can be determined using Lagrangian mass transport (Arzani et al., 2014, 2017). By this way, the particles in the blood flow can be tracked and their interaction with AAA wall can be modeled using convection, diffusion, and reaction equations coupled with the computed flow field (Biasetti et al., 2012). These fluid-chemical models provide better insight to understand AAA and ILT pathophysiology.

### Setting Up and Solution of the Solid Domain via FEA

#### Defining Material Constants

AAA wall has hyperelastic (i.e., non-linear stress-strain behavior), viscoelastic (i.e., time varying response due to relaxation) and anisotropic (i.e., direction dependent) material properties due to its layered and fiber-oriented structure (Vande Geest et al., 2006a,b). In order to determine the wall deformation and stress in a reliable manner, constitutive equations are needed to model wall stress-strain behavior. As a first approximation, AAA wall can be modeled as a linearly elastic, homogeneous medium using elastic modulus of 2.7 MPa, Poisson's ratio of 0.45, and mass density of 2,000 kg/m^3^ (Di Martino et al., 2001). For improved accuracy, non-linear stress-strain behavior should be taken into account using hyperelastic models (Scotti et al., 2008). Raghavan and Vorp (2000) obtained an experimental fit for non-linear stress-strain curve of AAA wall. Experimental data can be represented using Mooney-Rivlin hyperelastic material model considering a strain energy density function (*W*_*s*_) given in Equation (11) (Rivlin and Saunders, 1951).

(11) $$\begin{array}{l} W_{s}=c_{10}\left(I_{1}-3\right)+c_{01}\left(I_{2}-3\right)+c_{20}{\left(I_{1}-3\right)}^{2}+\\ c_{02}{\left(I_{2}-3\right)}^{2}+c_{11}\left(I_{1}-3\right)\left(I_{2}-3\right)+c_{30}{\left(I_{2}-3\right)}^{2} \end{array}$$

In Equation (11), *I*_*i*_ is the *i*th invariant of the left Cauchy-Green tensor, *c*_*ij*_ is the material parameter for fitting the experimental data. Chandra et al. (2013) used second order Mooney-Rivlin approach for modeling the AAA wall and ILT as given in Equations (12,13), respectively.

(12) $$\begin{array}{l} W_{wall}=c_{10}\left(I_{1}-3\right)+c_{20}{\left(I_{1}-3\right)}^{2} \end{array}$$

(13) $$\begin{array}{l} W_{ILT}=c_{01}\left(I_{2}-3\right)+c_{02}{\left(I_{2}-3\right)}^{2} \end{array}$$

Considering the population averages (Raghavan and Vorp, 2000; Vande Geest et al., 2006a,b), material parameters in Equations (12) and (13) are determined as *c*_10_ = 17.4 N/cm^2^, *c*_20_ = 188.1 N/cm^2^, *c*_01_ = 7.98 N/cm^2^, and *c*_02_ = 8.71 N/cm^2^. Mass densities of AAA wall and ILT are assumed to be 1.2 and 1.1 g/cm^3^, respectively.

#### Boundary Conditions in Solid Domain

In general, AAA tissue is isolated by applying zero translation and zero rotation fixed on the inlet and outlet of the solid domain (Scotti et al., 2005). The surrounding branch arteries of aorta produce tethering effect on the AAA model. This tethering effect is reflected to the wall either by using previously described zero displacement and rotation fixed on the inlet and outlet boundaries; or alternatively, an axial stretch can be defined on these solid inlet and outlet boundaries as suggested by Tang et al. (2005), since arterial wall is physiologically under tension in reality (Holzapfel et al., 2000). Scotti et al. (2008) applied 5% axial stretch on the inlet and outlet boundaries of the solid for modeling the tethering effect.

In addition to inlet and outlet boundaries, intra-abdominal pressure and contact of AAA with the surrounding tissues and organs can also be considered for defining boundary conditions (BC) in solid domain. Scotti et al. (2008) applied intra-abdominal pressure of 12 mmHg (Hinnen et al., 2005) on the outer AAA wall. In reality, AAA is located along the spinal column and there is a certain physical contact between AAA and spinal column which provides additional support on the posterior wall. This can be modeled with an additional BC on the posterior wall by fixing the contact surface with zero displacement (Scotti et al., 2008).

If solid FEA model is not coupled with a flow domain, then intraluminal pressure BC is applied on the inner AAA wall as a steady or transient load. This analysis is also known as computational solid stress (CSS) approach. In case of a FSI simulation, in which the solid FEA model is coupled with a flow domain, inner AAA wall is set as a FSI boundary surface.

### Setting Up and Solution of the Fluid Domain Via Computational Fluid Dynamics

#### Modeling Non-Newtonian Fluid Viscosity

Computational fluid dynamics (CFD) simulations are performed to numerically determine the flow variables such as flow velocity, pressure and WSS by modeling the fluid viscosity. Newtonian fluids have constant viscosity, but for non-Newtonian fluids such as blood, the viscosity changes as function of shear rate. For large arteries, the shear strain rate in the flow exceeds 50 s^−1^ and the viscosity demonstrates nearly constant behavior depending on the high shear rate (Young, 1979). Therefore, modeling the blood as a Newtonian fluid is a common approach for the AAA flow. Recently, Arzani (2018) reported that blood residence time affects the viscosity and traditional non-Newtonian models may exaggerate the shear-dependent viscosity behavior of blood, thus it is recommended to use Newtonian models if minor portion of AAA is accompanied by high blood residence time.

Blood mass density is usually taken as 1.05 g/cm^3^ with a constant dynamic viscosity (μ) of 0.035 Poise (Chandra et al., 2013). Khanafer et al. (2006) compared Newtonian and non-Newtonian fluid models for the same AAA geometry and stated that the maximum pressure and maximum WSS differences were 2.53 and 26.7%, respectively. The higher difference in maximum WSS is due to the effect of near-wall turbulence which is underestimated by Newtonian models. Non-Newtonian behavior of blood can be modeled using Carreau-Yasuda viscosity model parameters given in Equation (14).

(14) $$\begin{array}{l} \eta \left(\dot{\gamma }\right)=\eta_{\infty }+\left(\eta_{0}-\eta_{\infty }\right){\left[1+{\left(\lambda \dot{\gamma }\right)}^{a}\right]}^{\left(n-1\right)/a} \end{array}$$

In Equation (14), $\dot{\gamma }$ is shear rate, η is shear rate dependent viscosity, η_∞_ is viscosity at high shear rate, η_0_ is viscosity at low shear rate, and λ, *a*, *n* are empirical constants. By approximating the blood viscosity measurements of Thurston (1979), empirical parameters yield as η_∞_ = 0.00476 Pa s, η_0_ = 0.0519 Pa s, *a* = 0.409, *n* = 0.191, and λ = 0.438s.

#### Fluid Domain Boundary Conditions

**Figure 4:**
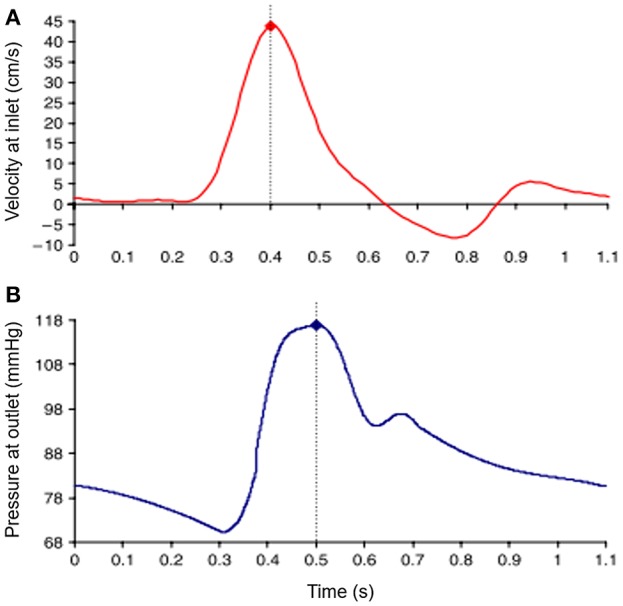
Waveforms of applied boundary conditions at the inlet and the outlet of the fluid domain. Dash lines show the moment of peak values in the waveforms. **(A)** Sample inlet flow velocity profile. **(B)** Sample outlet pressure profile [The figure is adapted from Scotti et al. (2008) and used with permission].

Inlet and outlet BC are defined considering the physiological pulsatile flow. Commonly, time-varying flow velocity given in Figure 4A is defined at the inlet of fluid domain and time-varying intraluminal pressure given in Figure 4B is prescribed at the outlet (Scotti et al., 2008). No slip BC is applied on the wall considering the viscous blood flow.

The time-dependent inlet flow rate given in Figure 4A can be applied considering different velocity profiles. Applying plug velocity profile is the simplest approach where the axial flow velocity is uniform through the inlet boundary surface (Bluestein et al., 2009). Alternatively, fully developed velocity profile can be used considering Womersley profile which has zero velocity on the wall and has a parabolic distribution with maximum flow velocity at the midpoint of the inlet surface, which is more realistic due to including the effect of viscous boundary layer (Womersley, 1955; Papaharilaou et al., 2007). To enhance the accuracy of the model, patient-specific inlet velocity profiles can also be applied by obtaining realistic profiles non-invasively using phase-contrast magnetic resonance images (PC-MRI) (Kose et al., 2006).

After determining patient-specific inlet BC, distribution of mass flow rate to the branches of mesenteric, renal, and iliac arteries should also be considered, since they are significantly affecting the mass flow rate at the outlet of AAA. One-dimensional arterial tree model can be used to determine appropriate inflow and outflow conditions to better reflect the reality for biological relevance (Formaggia et al., 2003; Wolters et al., 2005). Les et al. (2010) modeled the effect of branch arteries at the downstream vasculature using three-element Windkessel model considering the capacitance, proximal resistance and distal resistance at the downstream. Using PC-MRI scanning, mean flow rates at supraceliac and infrarenal locations are measured as 3.51 and 1.31 L/min, respectively. The difference of 2.2 L/min is distributed to the arterial branches between supraceliac (1 cm above celiac artery) and infrarenal (1 cm below the most distal renal artery) levels (Les et al., 2010). It is reported that using zero-pressure outlet BC is not sufficient, and Windkessel BC provides more realistic flow and pressure features.

For determining patient-specific outlet pressure BC, a catheter can be placed inside AAA sac, however this invasive procedure is not preferred by clinicians during AAA repair (Chandra et al., 2013). Alternatively, a non-invasive method can be applied to obtain patient-specific outlet pressure. van ‘t Veer et al. (2008) compared non-invasive brachial cuff blood pressure measurements with invasive catheter pressure measurements inside AAA sac. Brachial cuff pressure measurements resulted in 5% underestimation for systolic phase, and 12% overestimation for diastolic phase compared to the intraluminal pressure in AAA. Using these correlations, a patient-specific estimation of fluid outlet pressure can be predicted non-invasively.

### Solution and Mesh Independence for Solid and Fluid Domains

**Figure 5:**
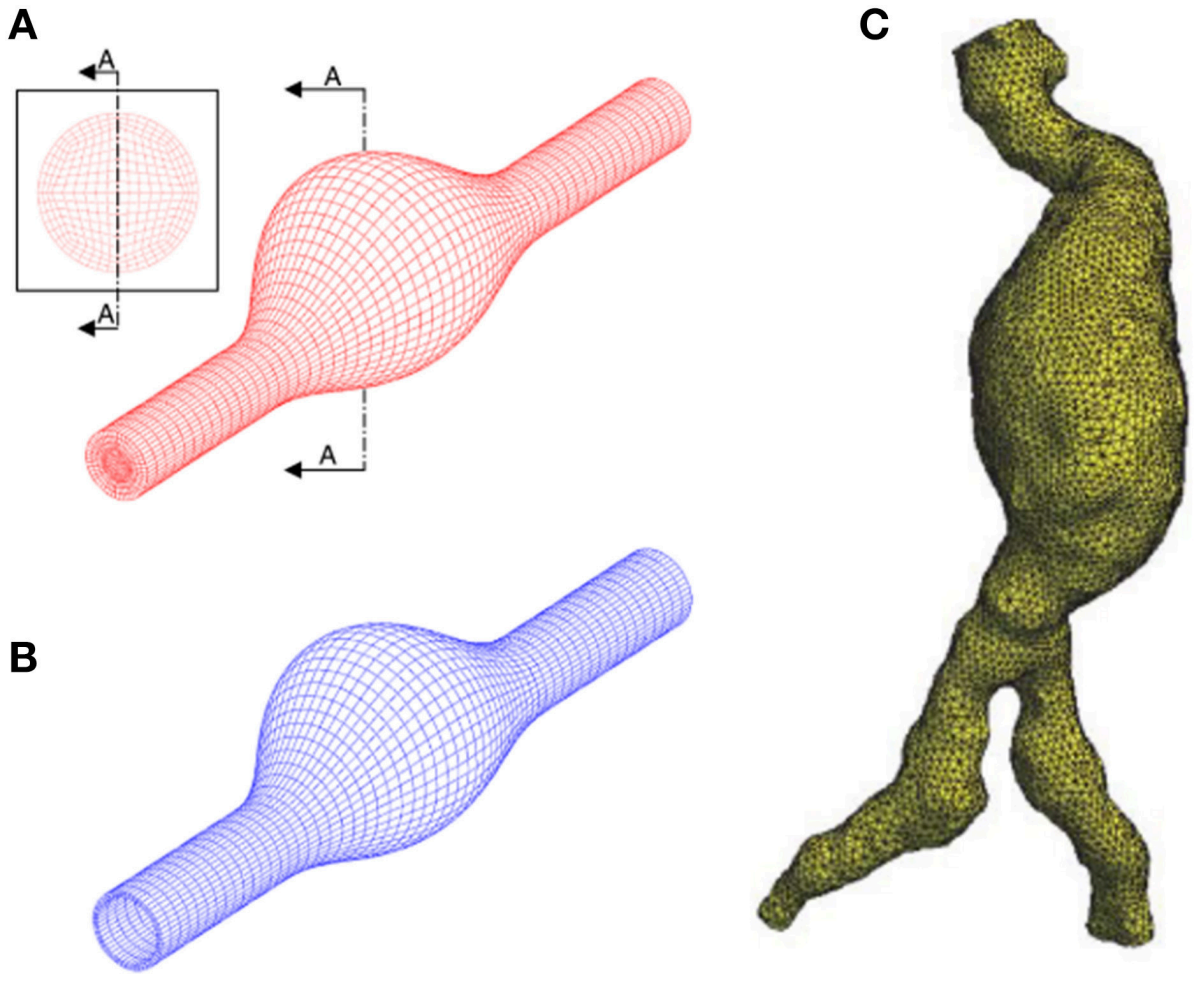
Discretization of the problem domain using finite element meshes. **(A)** Structured hexahedral fluid mesh using idealized AAA model. **(B)** Structured hexahedral solid mesh using idealized AAA model. **(C)** Unstructured tetrahedral fluid mesh using patient-specific AAA model. [**A,B** are adapted from Scotti et al. (2008) and used with permission, **(C)** is adapted from Wolters et al. (2005) and used with permission].

Solid and fluid domains are spatially discretized using a generated mesh composed of tetrahedral, hexahedral or polyhedral elements as given in Figure 5. In fluid domain, mesh density should be relatively higher in the boundary layers close to the wall and in regions that are expected to have high velocity gradients as well. Obtaining a mesh-independent solution is a requirement prior to the in depth analysis of AAA hemodynamics. Typically, if a relative difference of 2% is achieved for variables obtained by different mesh sizes, the results can be considered as mesh-independent (Kelsey et al., 2016). The quality of CFD mesh resolution can also be checked using the minimum Kolmogorov length scale. For large eddy simulation approach, the ratio of mesh element size to the Kolmogorov length scale should be at most 20 and 40 for fine and coarse fluid meshes, respectively (Celik et al., 2009).

In order to check the mesh-independency of results, solution of first three cardiac cycles should be ignored. Because, periodically converged results are generally obtained after the third cycle. A time step of 0.001 s (~1/1,000th of cardiac cycle) is suitable for obtaining unsteady solutions (Les et al., 2010). If stability problems arise in the analysis, the time step can be decreased to achieve convergence. Courant number (*Co* ≡ *u*(*t*)/*x*) should be equal or lower than one for the increased stability. The Courant number shows how much information traverses (i.e., Flow velocity, *u*) the length of a mesh element (*x*) within a time step (*t*).

### Fluid-Structure Interaction (FSI)

To simulate deformation of AAA tissue under the effect of blood hemodynamics accurately, FSI approach needs to be adapted. This is because of the strong interactions between flowing blood and vessel walls. Blood flow generates unsteady forces on vessel walls that causes deformation of the walls. These deformations in turn influence blood flow patterns. Therefore, vessel wall behavior cannot be predicted accurately if these counter interacting forces are ignored. The commercial software packages such as ANSYS, ADINA, ABAQUS, COMSOL are commonly used for FSI modeling.

FSI modeling can be performed using three different numerical approaches, which are 1-way uncoupled (also known as FEA or CSS), explicit 2-way coupled and implicit 2-way coupled methods. 1-way uncoupled method is a static investigation where spatially uniform or non-uniform intraluminal pressure load is applied to the inner AAA wall without considering the effects of dynamic flow and only provides solid domain parameters such as wall stresses and wall displacements. In a related study (Scotti et al., 2008), it is stated that 1-way uncoupled approach underestimates the maximum wall stress due to neglecting the hemodynamic effects. To have a better understanding, FSI analysis should be performed using 2-way coupled methods. In a numerical investigation, 1-way uncoupled and 2-way coupled FSI simulations are compared using the same patient-specific AAA model and it is reported that 1-way uncoupled FSI resulted in 14, 4, and 18% difference in peak principle stress (σ_1_), principle strain (ε_1_), and WSS, respectively (Chandra et al., 2013).

In 2-way coupled methods, a FSI boundary is defined between the blood and AAA wall. On this FSI boundary surface, solutions of solid and fluid domains are coupled considering displacement compatibility and traction equilibrium given in Equations (15,16) (Bathe et al., 1999). On FSI boundary, solid and fluid displacement vectors are denoted by **d**_*s*_ and **d**_*f*_; solid and fluid unit normal vectors are denoted by ${\hat{\text{n}}}_{s}$ and ${\hat{\text{n}}}_{f}$, respectively.

(15) $$\begin{array}{l} {\text{d}}_{s}={\text{d}}_{f} \end{array}$$

(16) $$\begin{array}{l} \tau_{s}▪{\hat{\text{n}}}_{s}=\tau_{f}▪{\hat{\text{n}}}_{f} \end{array}$$

Two-way coupled FSI can be performed using explicit or implicit approaches depending on the level of physical interaction between the fluid and solid. When a strong interaction exists as present in AAA, explicit approach will be insufficient, and implicit approach should be preferred for enhanced accuracy (Amindari et al., 2017). For implicit approach, there are two options as iterative implicit or fully coupled. In fully coupled implicit method, all governing fluid and solid equations are solved simultaneously, requiring high computational memory and leading to an excessive solution time. However, iterative implicit approach requires less computational power, and consists of a number of coupling iterations until reaching interaction convergence at each time step. Using smaller time steps lead to more stable results. Increasing the number of coupling iterations and using relaxation factors will help to overcome the stability problems.

### Recent Findings on Growth and Rupture Mechanics of AAAs From Computational Studies

It is now widely accepted that biomechanical factors play role in degeneration and eventual rupture of AAA tissue. There is controversy regarding which biomechanical parameters are important in rupture. Below we summarize the important findings by categorizing the effects of various parameters.

#### Effect of Wall Thickness and AAA Asymmetry

Scotti et al. (2005) investigated the effect of varying wall thicknesses by performing fully coupled FSI simulations using idealized AAA geometries based on medical images. The thickness of AAA wall has an average of 1.45 mm, but it can decrease to 0.23 mm near the rupture site (Raghavan et al., 2004, 2006). A variable thickness between 0.5 and 1.5 mm was distributed along the AAA wall as inversely proportional to the cross-sectional diameter. When variable wall thickness model was compared with a uniform wall thickness (1.5 mm) model, it was observed that peak wall stress was increased by four-times for the variable wall thickness case.

The same research group also investigated the effect of AAA asymmetry (Scotti et al., 2008). Different levels of asymmetry were modeled using idealized geometries with the same patient-specific inlet velocity BC. For the most asymmetric model with β = 0.2 (see Equation (1) for definition of β), AAA diameter expanded by 15.2% at the peak systolic pressure. When an axisymmetric (β = 1) AAA model is considered, the diameter expansion is observed as 12.8%, implying that increasing asymmetry resulted in higher AAA deformation and therefore higher peak wall stress on the wall. As AAA becomes more asymmetric, location of peak wall stress shifted from anterior to the posterior wall.

#### Effect of Proximal Neck and Iliac Bifurcation Angles

Drewe et al. (2017) performed FSI simulations to investigate the effects of proximal neck and lateral iliac bifurcation angles considering idealized AAA models. Recent morphological comparisons showed that AAAs with large iliac bifurcation angle have a lower rupture risk (Drewe et al., 2017). Proximal neck angle has less impact on AAA hemodynamics compared to the iliac bifurcation angle. When the iliac bifurcation angle increased from 30 to 150°, peak WSS increased more than 2-fold (from 2.91 to 6.19 Pa), peak von Mises wall stress increased about 30% (from 0.186 to 0.243 MPa) and ECAP decreased by 57% (from 11.41 to 7.25). Larger iliac bifurcation angle was more protective of ILT formation and AAA expansion due to provoking high WSS and low ECAP conditions (Drewe et al., 2017). However, excessive load on the iliac arteries with increased bifurcation angle may increase the risk of an iliac artery aneurysm initiation (Xenos et al., 2010).

#### Effect of AAA Diameter and Wall Stress

The maximum AAA diameter is the first indicator for the treatment. For the current practice, when maximum AAA diameter exceeds 5–6 cm or diameter growth rate is higher than 1 cm per year, open surgery or endovascular treatment methods are performed considering the life expectancy of the patient (Scott et al., 2002; Longo and Upchurch, 2005; Chandra et al., 2013). Canchi et al. (2018) performed a comparative FSI study, considering two patient-specific AAAs with maximum aneurysmal diameters of 3.5 and 7 cm. Maximum principle stresses were determined as 0.30 and 0.22 MPa for 3.5 and 7 cm AAA diameters, respectively, implying that size of AAA is not the sole determinant for the rupture risk. It is reported that maximum wall stress was 12% more accurate for predicting rupture compared to using maximum AAA diameter alone as an indicator (Fillinger et al., 2002, 2003).

**Figure 6:**
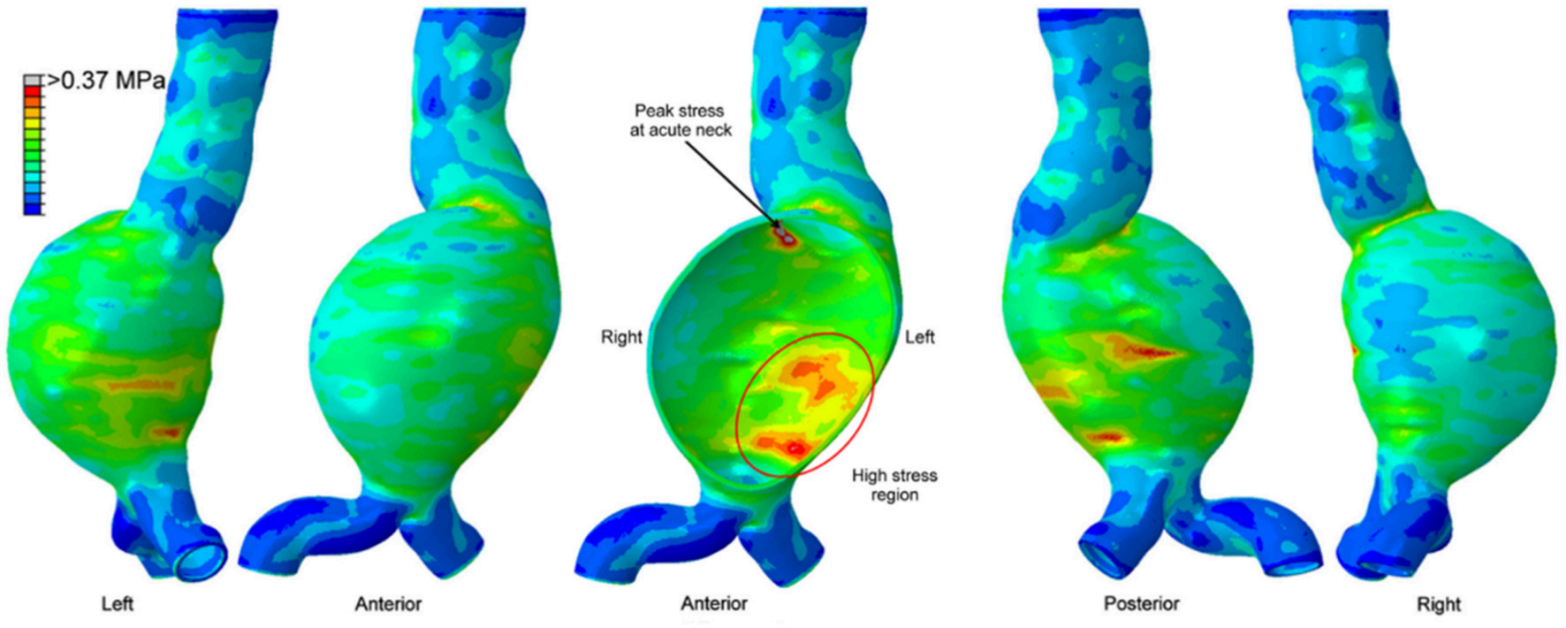
Von Mises wall stress contour plot from different views, on a patient-specific AAA geometry at peak systolic pressure. The middle figure is a sectional view. High stress region exists in the posterior wall, however the peak wall stress is observed at the proximal neck of the aneurysm [The figure is adapted from Doyle et al. (2014) and used with permission].

The maximum wall stress is generally observed at the transition of sac to neck of AAA wall as shown in Figure 6 (Chandra et al., 2013; Doyle et al., 2014). FEA investigations showed that peak wall stress on the posterior AAA wall was within the range of 290 to 450 kPa (Raghavan et al., 2000), while non-aneurysmal aorta had a peak stress around 120 kPa. Scotti et al. (2008) compared FSI and FEA approaches on the same AAA model, and showed that FSI resulted in 25% increased wall stresses (peak values between 275 and 398 kPa) compared to FEA. Chandra et al. (2013) also performed FSI and obtained relatively higher peak wall stresses between 750 and 870 kPa. The reason for the high peak wall stress was considered to be the non-uniform AAA wall thickness which could increase the stresses at low thickness areas.

#### Effect of WSS and OSI

WSS and OSI are important hemodynamic parameters in turbulent AAA flow. Les et al. (2010) investigated patient-specific AAA hemodynamics using high-resolution CFD simulations (about 8-million mesh elements), hypothesizing that physical lower limb exercise might decrease the growth rate of AAA, since exercise resulted in a high amplitude WSS pattern and lowered OSI on AAA wall. Moderate turbulence was observed in AAA during exercise, while resting conditions led to mild turbulence. Rest-to-exercise TAWSS changes were found to be statistically significant. For example, at supraceliac level, TAWSS at rest was 3.6 dyn/cm^2^, and it increased to 9.2 dyn/cm^2^ during the exercise. At mid-aneurysm level, TAWSS at rest was 7.3 dyn/cm^2^, while at exercise it was 21.7 dyn/cm^2^. OSI values at rest were 0.28 and 0.27 for supraceliac and mid-aneurysm locations, respectively. These OSI values decreased to 0.18 and 0.21 during the exercise.

**Figure 7:**
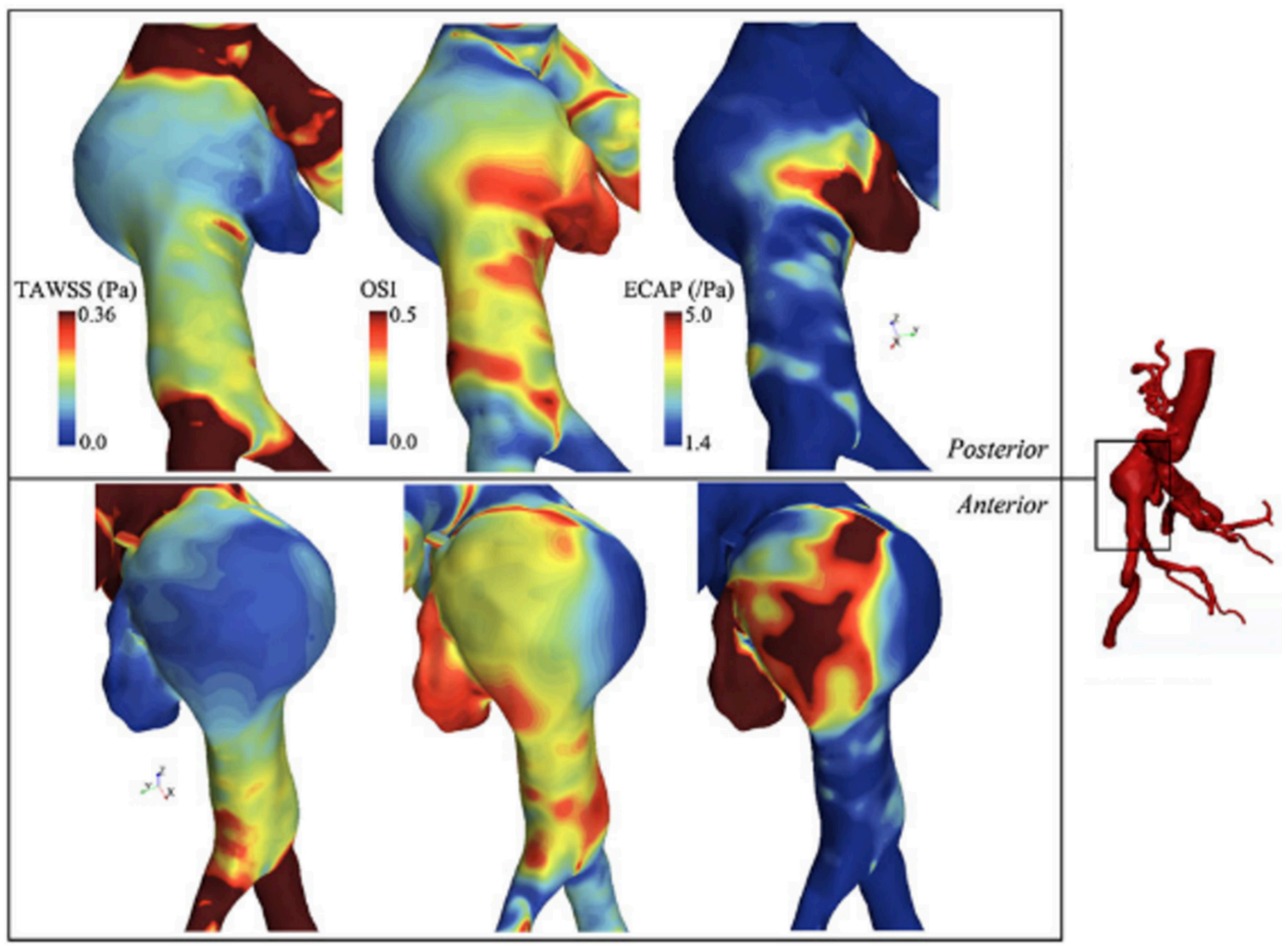
TAWSS (Time averaged WSS), OSI, and ECAP contour plots on a patient specific AAA model. ILT formation is more likely to be observed in high ECAP regions. Rupture risk and aneurysm growth rate increase at regions with high OSI and low TAWSS [The figure is adapted from Kelsey et al. (2016) and used with permission].

Qiu et al. (2018) performed CFD simulations using three ruptured and one non-ruptured patient-specific AAA models. The rupture sites were found to be near the fluid stagnation regions which have nearly zero WSS with high WSS gradients (WSSG). In Figure 7, the distribution of WSS and OSI are provided on a patient-specific model. Most researchers agree that the locations with low WSS, high OSI and high ECAP are prone to thrombus formation and have a higher risk of rupture (Les et al., 2010; Kelsey et al., 2016). On the other hand, in some studies reporting controversial results (O'Rourke et al., 2012; Arzani et al., 2014; Mohamied et al., 2015; Singh et al., 2018), it is stated that low WSS and high OSI regions do not coincide with thrombus deposition and atherosclerosis sites. Therefore, the exact effect of these hemodynamic parameters on the rupture mechanism is not yet fully understood.

#### Effect of Vascular Growth and Remodeling

Arterial growth and remodeling are investigated and modeled in several studies (Humphrey and Rajagopal, 2003; Watton et al., 2004; Gleason and Humphrey, 2005; Valentín et al., 2011, 2013; Humphrey and Holzapfel, 2012; Karšaj and Humphrey, 2012). In a recent study, Wu and Shadden (2015) presented a computational framework by coupling blood flow hemodynamics with vascular growth and remodeling (G&R). The wall is treated as a constrained mixture consisting of anisotropic collagen, elastin and smooth muscle fibers. Depending on the mechanical stimuli on the wall, the vessel has the ability of adaption for returning back to its homeostatic state by means of removal of old constituents and production of new ones. The lifespan of collagen is within 70–80 days (Wilson et al., 2013), where the flow simulation cycle has a time scale around 1 s. For computational efficiency, flow simulations only performed in cases when G&R caused severe change in geometry and boundary conditions, meaning that the last flow simulation results were used for all time in between until a new one is performed. The deviations of wall tension and WSS were used as the influencing factors for mass production rate on the vessel to return its homeostatic state. WSS changed about one order of magnitude in the aneurysm site and contributed to G&R. Stresses on the wall converged to homeostatic values after 110 G&R time steps (equivalent to about 800 days) and significant wall expansion was observed at the entire mass loss introduced regions.

#### Effect of Surgical and Endovascular Treatment Methods

Sughimoto et al. (2014) evaluated AAA surgery by comparing pre-operative and post-operative hemodynamics on CT-based geometries. AAA was replaced with a 30 mm straight graft under cardiopulmonary bypass. They proposed a novel parameter of pulsatile energy loss index (PELI) which evaluates the energy loss between inlet and outlet of interested artery. After AAA treatment, PELI decreased from 0.986 (pre-operative value for the whole aorta) to 0.820 (see Sughimoto et al. (2014) for calculation of PELI), indicating that the grafting procedure improved the energy efficiency of blood flow delivery and reduced the left ventricle afterload. For a young healthy adult, PELI was measured as 0.0215, which was significantly low when compared to the AAA patient.

Casciaro et al. (2018) performed CFD simulations before and after two different endovascular surgical treatments of AAA. The Nellix endograft (Endologix, Irvine, California) proposed a method of AAA treatment based on endovascular aneurysm sealing (EVAS). For conventional endovascular aneurysm repair (EVAR), endografts have proximal fixation mechanism. The lateral neck angle (Kandail et al., 2015) and implantation position (Raptis et al., 2017) of the endografts alter the hemodynamics of EVAR. On the other hand, EVAS consists of two stents which are separately engaged to left and right common iliac arteries. Expandable endobags surround the stents and provide continuous sealing along the internal surface of the aneurysm (Böckler et al., 2015). Due to significant anatomic modifications after EVAS, 2-fold pressure increase was seen at the level of renal arteries. In addition, the peak flow velocity inside EVAS endograft stents was 60 cm/s, which was three-times higher than the peak blood flow velocity after EVAR treatment. This 3-fold increase in peak flow velocity after EVAS, also resulted in 60% higher WSS compared to EVAR (Casciaro et al., 2018).

## Experimental Investigation of Hemodynamics of AAAs

AAA hemodynamics is quite complex primarily due to irregular shape, flexible arteries, turbulent flow, and non-Newtonian behavior of blood. As computational methods have widely been used in investigation of hemodynamics and mechanical behavior of arterial tissue, experimental techniques are also utilized in characterization of flow dynamics through AAAs. Both approaches are crucial and complement each other with offering in depth analysis where the corresponding results depend on the assumptions in computational models and simplifications in experimental methods.

Various qualitative and quantitative flow measurement techniques have been utilized for experimental investigation of AAAs. In early studies, qualitative flow visualization techniques have been widely implemented, in which using localized injections, the patterns of dye as streaklines were generated (Ku et al., 1989). This simple method provides insights on overall behavior of flow structure in the region of interest. In terms of detailed velocity information in the AAAs, non-intrusive, and quantitative techniques such as MRI and Doppler Ultrasound Imaging (DUS) are appropriate for detailed anatomical analysis by means of tomographic slices. In MRI based system encoding the flow velocity is obtained by means of the changes in MR signal phase along a magnetic field gradient (Wang et al., 2016). Moreover, laser-based techniques including Laser Doppler Anemometry (LDA) and Particle Image Velocimetry (PIV) are also implemented for detailed velocity information. In LDA, pointwise velocity measurements are performed by means of Doppler effect using two laser beams for each velocity component. This method has very high spatial and temporal resolutions (~kHz) and allows to measure reverse flow and turbulent fluctuations, which is quite critical for understanding the growth and the rupture mechanics of AAAs (Yip and Yu, 2002). In PIV, two component velocity information on 2-D plane is obtained at relatively lower temporal resolution (~Hz) using two laser sheets and an advanced camera, where in stereoscopic PIV three-velocity components are obtained on 2-D plane using two cameras. This method is quite effective in understanding the key parameters of disturbed hemodynamics of AAAs since it provides detailed velocity field information and therefore have widely been used recently (Deplano et al., 2013; Chen et al., 2014). For the quantification of three-dimensional velocity field, PIV offers two recent state of the art versions: Tomographic PIV and Holographic PIV, which are expected to appear in the studies of disturbed hemodynamics through AAAs in near future. Tomographic PIV has recently been applied to the hemodynamics of intracranial aneurysms (Roloff et al., 2017, 2018).

Experimental studies on blood flow inside AAAs is primarily categorized and populated in two major groups: the properties of the aorta phantom and conditions of the flow. Major consideration for the aorta phantom are elasticity of arterial wall and geometry of the aorta (change in wall diameter, aneurysm shape, artery bifurcation, etc.) whereas major considerations in flow conditions, include flow waveform (steady or physiological), type of working fluid (Newtonian, non-Newtonian), and pulse rate (exercise or resting). Considering the overall flow structure in aorta and AAAs, fully developed flow at the entrance of the AAA expands and creates counter rotating recirculation along with a jet flow at the center, which are due to the adverse pressure gradient imposed at the aneurysm bulge. Location and strength of the recirculation vortex primarily depend on the inlet flow waveform, bulge shape, and elasticity of arterial wall. For the steady inlet flow condition, which is not realistic, the recirculating vortex is larger in extent in average and located closer to downstream of the bulge, whereas in physiological flow condition recirculation region is alternating and moving back and forth due to periodic flow condition. It is also indicated that the spatial extent, the location, and the strength of the recirculation vortex are significantly affected by the bulge shape and the wall rigidity (Egelhoff et al., 1999; Yu, 2000; Deplano et al., 2007; Meyer et al., 2011). Flow structure inside the AAAs varies significantly according to these considerations, as reported by many experimental studies (Egelhoff et al., 1999; Yu, 2000; Deplano et al., 2007; Meyer et al., 2011).

Therefore, because of the complexities in geometry and flow conditions, the experimental set up for AAA investigations need to be designed to mimic natural realistic *in-vivo* conditions within AAA as close as possible to obtain accurate results. A typical experimental set up for the analysis of hemodynamics contains flow circulatory system including pump, piping, pressure compliance, test section that contains artery model, blood mimicking fluid, and flow measurement systems as briefly mentioned previously such as MRI, LDA, or PIV. In this part of the paper, the components of experimental set up and the techniques that are used widely in literature are explained in detail, and the results of recent experimental studies are discussed.

### Flow Circulatory System

**Figure 8:**
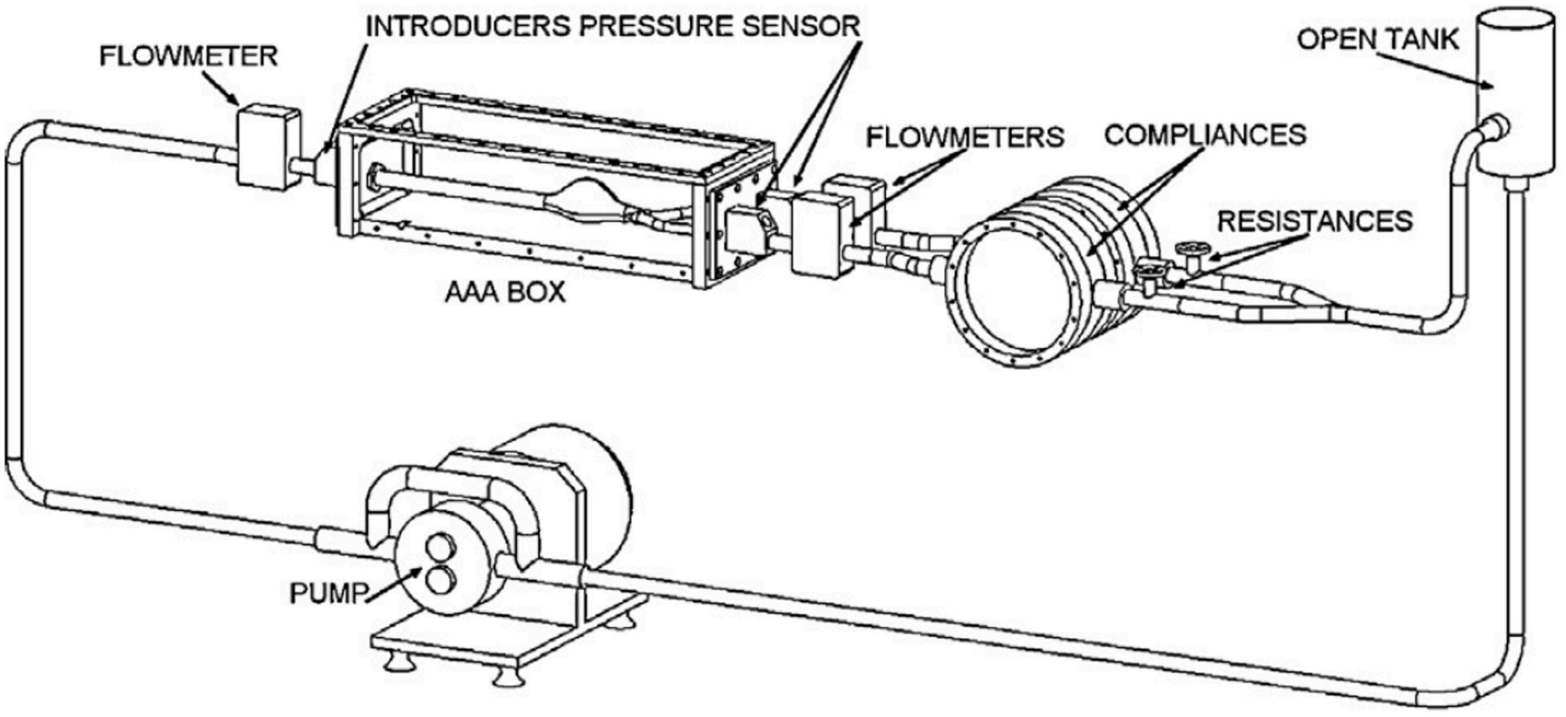
Circulatory flow loop model for flow measurement system of abdominal aortic aneurysm containing tank, physiological flow generator, flowmeters, pressure sensors, compliances, and AAA box [The figure is adapted from Deplano et al. (2013) and used with permission].

In cardiovascular biomechanics, it is difficult to measure hemodynamic quantities clinically due to the constraints of the system. Conducting *in vivo* animal studies are also very challenging because of the geometrical complexities and ethical issues. Therefore, in recent years, simulating *in vivo* conditions by using computational models or experimental setups become very popular. *In vitro* studies can be performed by generating the physiological flows through anatomical geometries of interest to simulate exact *in vivo* conditions in laboratory test setup; i.e., flow rates and pressure at the inlet and the exit, geometry of the artery and working fluid of a specific artery should be replicated physiologically. In Figure 8, an example of a test setup is presented. The main component of such a setup is the flow source, which is generally a pump, supplying a fluid flow into downstream. At the downstream, there are resistances to simulate physiological pressures. This is generally accomplished by using compliance chambers; in other words, vascular simulators to mimic realistic arterial pressures for specific arteries. Besides mimicking the physiological flow conditions, arterial phantoms which are desired to be studied should also be modeled and manufactured (Doyle et al., 2008, 2009b; Corbett et al., 2009). Finally, a blood mimicking fluid should be selected that matches biomechanical behaviors of blood.

#### Pump

Blood flows through arteries in an unsteady manner, which is characterized by a rhythmic repeatability and called pulsatile, i.e., such flows repeat themselves with a certain frequency. The pulsatile physiological flow profile, is presented in Figure 4, and has a complex waveform. In earlier studies, although it is far from the exact physiological conditions, researchers use constant flow rate in hemodynamic analyses. This is primarily due to the simplicities both in generating the steady flow in a circulatory loop and in performing flow measurements in such systems. In that sense, many experimental studies have been conducted at steady flow conditions using different type of flow generators, such as variable speed centrifugal or gear pumps (Ku et al., 1989; Asbury et al., 1995; Bluestein et al., 1996; Boutsianis et al., 2008), steady submersible pumps (Chen et al., 2014, 2015) or sometimes, by only using the effect of gravity, which requires placing a head tank (O'Rourke and McCullough, 2010).

However, a better description of hemodynamics of AAA closer to the physiological conditions is needed for the complete understanding of growth and rupture behavior of AAA. Indeed, comparison of steady and pulsatile flow conditions reveals important differences in hemodynamics which are also influenced by other system parameters including wall rigidity and AAA bulge shape (Yu, 2000). For example, with steady flow conditions, a vortex ring is localized at the distal end of the bulge, creating oscillations in wall shear stresses proximal to the corresponding localized region. On the other hand, with pulsatile flow case, the vortex ring is not localized and appears at the proximal site at the early systolic phase proceeding through the downstream throughout the cardiac cycle (Yu, 2000). This means that extrapolating steady flow results in extreme conditions might underestimate certain key parameters in physiological flow conditions. Considering the steady flow generators, centrifugal pumps, gravity-driven systems, and gear pumps have been commonly used. Later on, researchers have adapted various components to generate pulsatility at the downstream of these steady flow generators, including computer controlled valves (Egelhoff et al., 1999), piston-cylinder arrangements (Stamatopoulos et al., 2010, 2011), and gear pumps (Mechoor et al., 2016). In addition, commercial pumps which can generate either steady or pulsatile flow waveforms for several arteries are also available. All these alternatives for pumps are discussed along with addressing the pros and cons of each of them.

##### Steady flow generators

*Centrifugal pumps.* The most widely used flow generators are steady flow pumps, where the centrifugal pumps (Asbury et al., 1995; Bluestein et al., 1996) and submersible pumps (Chen et al., 2014, 2015) are generally used for that purpose. Centrifugal pumps are cost effective and are easily available in market with a very wide product range, however the flow rate is highly affected by the pressure drop, and thus, is not very convenient for hemodynamic analyses.

*Gear pumps.* Gear pumps are positive displacement pumps and are convenient for providing quite stable and constant flow rates at broad range of pressure drop conditions. In earlier studies, variable speed gear pumps have been employed in hemodynamics studies to generate steady flow conditions (Asbury et al., 1995; Boutsianis et al., 2008).

*Gravity-driven systems.* In gravity driven systems, the effect of gravity is used as the driving force with locating a head tank of fluid at certain elevation above the test section to maintain pressure and velocity distribution in AAA. With the effect of gravity, steady mean flow can be generated, but in order to maintain the physiological flow pattern, an additional equipment such as computer-controlled valves (Egelhoff et al., 1999) or piston-cylinder arrangements (Yu, 2000; Stamatopoulos et al., 2010, 2011) are needed to be used at the downstream.

##### Pulsatility generators

In order to generate pulsatility, additional computer-controlled equipment should be adapted to circulatory systems. For this purpose, the most common integrated systems include computer-controlled valves, piston-cylinder arrangements, or gear pumps.

*Valves.* In general, centrifugal pumps are adapted only for steady circulations, where in a very few studies, a flapper nozzle valve is adapted to the flow loop to produce pulsatility (Moore et al., 1992; Moore and Ku, 1994). In general, the desired waveform including physiological pattern with the reverse flow cannot be maintained properly in centrifugal pump driven systems. An application of valves in physiological flow generation is utilized by Egelhoff et al. (1999), where a gravity driven pump with a head tank kept 234 cm above the AAA test section to provide desired entrance flow conditions and a computer controlled diverter valve at the downstream of AAA model have been used to generate the pulsatility. The flow waveform for the corresponding setup is quite similar to realistic case, but deviates from the exact physiological waveform. A similar gravity driven set up with computer controlled rotating spherical valve downstream of the test section can also be used (Nikolaidis and Mathioulakis, 2002). Relatively enhanced version of the gravity driven flow generator with valves has been developed by Peattie et al. (2004), where two computer-controlled valves, one in the forward direction while the other is in the retrograde direction, were embedded to the system and more realistic waveform with backflow region was obtained with the improved control mechanism compared to Egelhoff et al. (1999) and Nikolaidis and Mathioulakis (2002).

**Figure 9:**
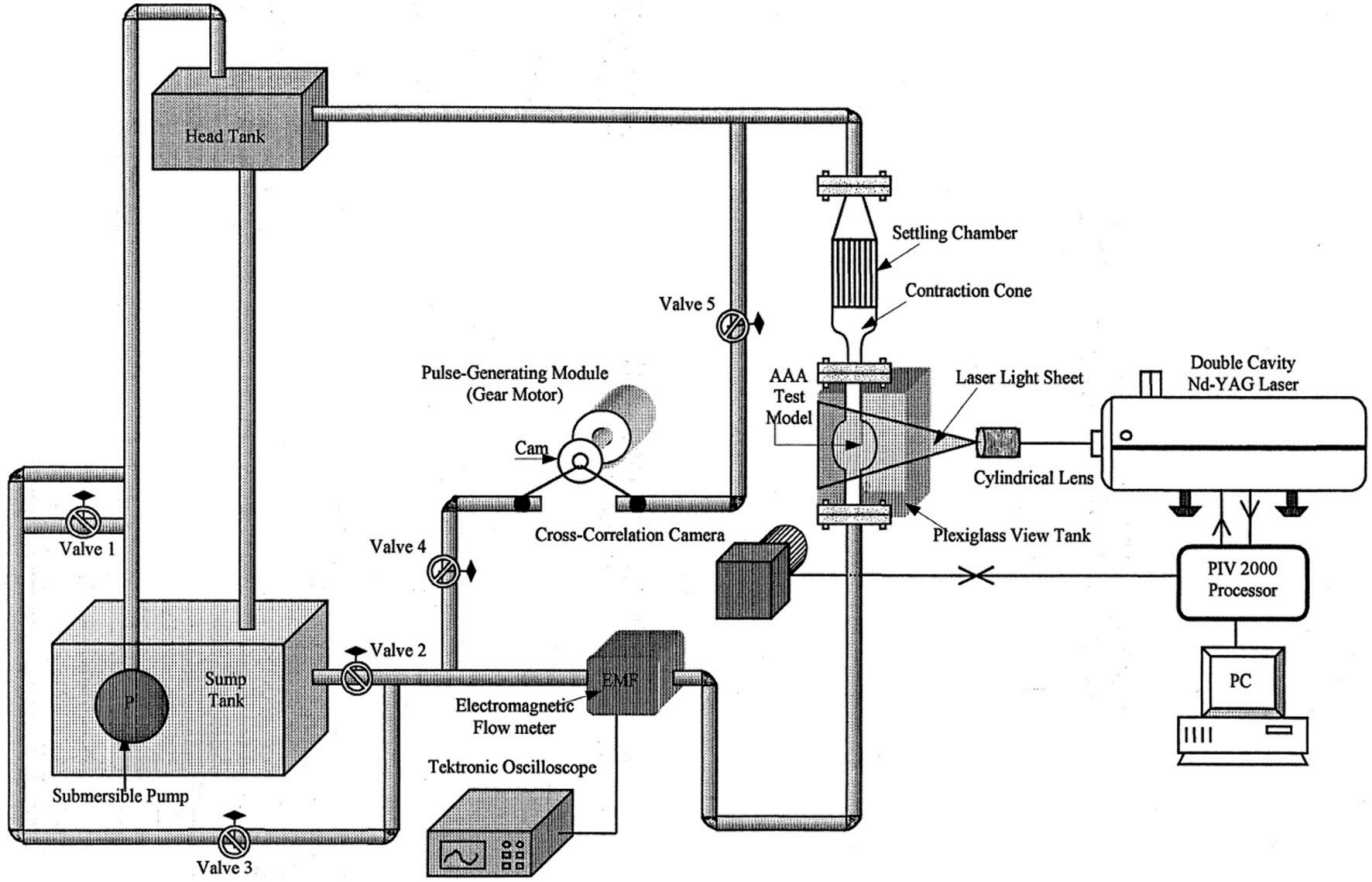
Experimental test set up for AAA test model. The mean flow is generated by a head tank and pulsatility is added by piston cylinder assembly fitted with a circular cam, which is driven by a DC gear-motor [The figure is adapted from Yu (2000) and used with permission].

**Figure 10:**
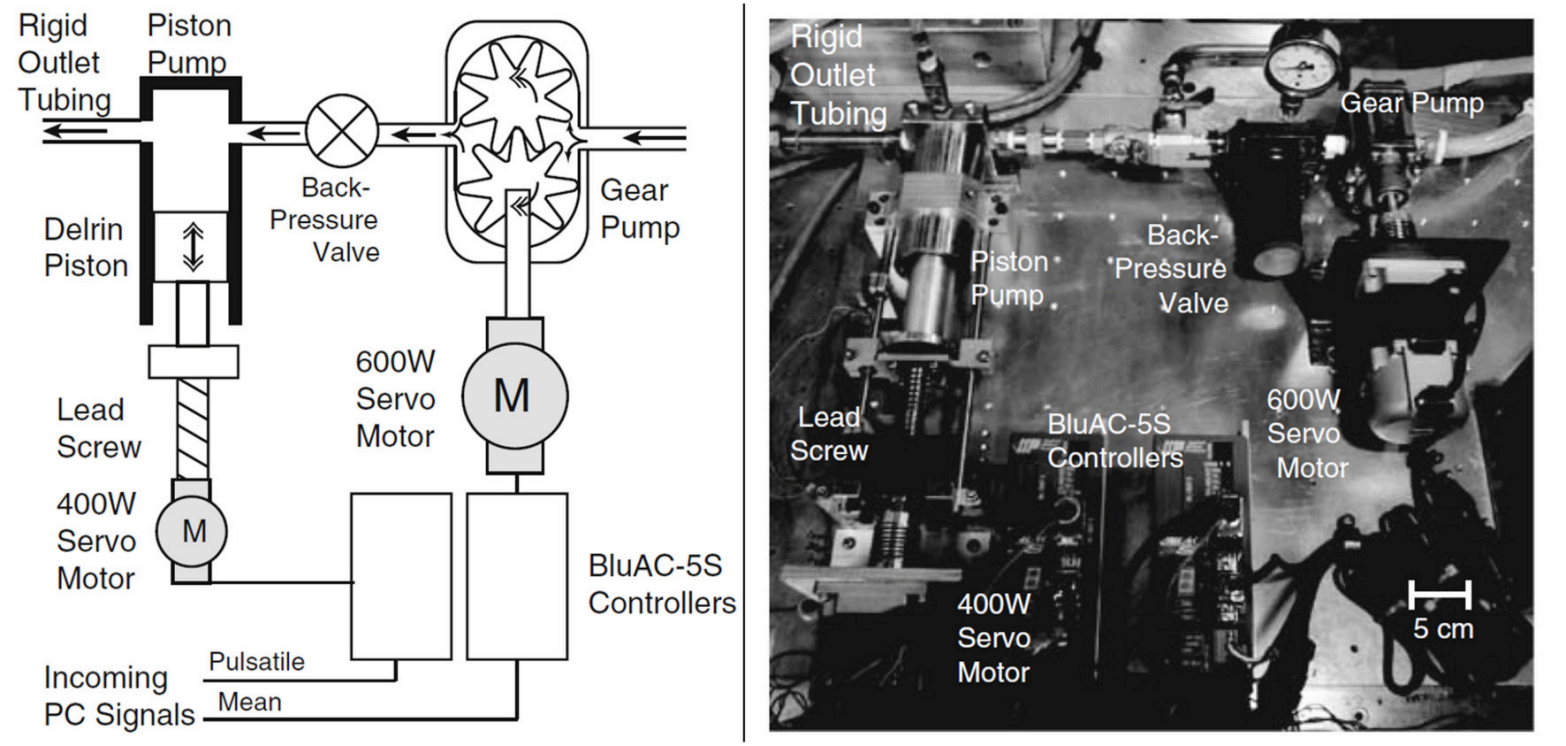
Sketch and picture of flow circulatory system using gear pump and piston-cylinder arrangement with lead screw as flow generator. Steady flow is supplied by gear pump, while piston and servo motor combination generates the pulsation [The figure is adapted from Tsai and Savaş (2010) and used with permission].

*Piston-cylinder arrangements.* In piston-driven pumps, a piston is used as linear actuator to provide periodicity to the mean flow that is generated by a gear pump or a head tank located at a certain elevation above the test section, as explained previously. Piston-driven pumps serve better performance in terms of controllability to generate desired flow rate compared to previous orientations, but still physiological waveform, especially backflow regions, cannot be generated properly. In Figure 9, a test rig with a piston-driven pump is presented (Yu, 2000). The mean flow in the test section is generated by a head tank, which is fed by a submersible pump. Pulsatility is generated by means of a pulse generating module, which contains a DC gear-motor fitted with the circular cam of a small piston-cylinder assembly. DC gear-motor drives the piston by means of the circular cam to create necessary pulsating flow conditions (Yu, 2000; Yu and Zhao, 2000). The flow waveform generated in such a flow loop is periodic in sinusoidal form but again not physiological. To obtain more realistic flow waveforms, in later studies, a variable speed electrical motor is used to drive circular cam of linear reciprocating piston (Yip and Yu, 2001, 2002; Stamatopoulos et al., 2010, 2011). In some orientations, motors drive the reciprocating pistons with screw mechanisms such as rack and pinion and lead screw, rather than circular cam (Duclaux et al., 2010; Ene et al., 2011; Morris et al., 2013; Wang et al., 2016). In Figure 10, an example for piston with lead screw mechanism is shown (Tsai and Savaş, 2010). In that case, a gear pump provides the steady mean component of the desired flow waveform and piston arrangement provides the oscillatory component of the flow waveform with the help of a motor driven lead screw. The gear pump is preloaded with a back-pressure valve at its discharge, providing that the gears remain in constant contact to prevent reverse flow. Although the system is capable of generating different pulsatile waveforms, great deviations from physiological cases are reported, especially for the regions of steep changes in the waveform (Tsai and Savaş, 2010). Sometimes two-sided cylinders are also employed with such screw mechanisms. In such a case, a computer controlled motor driven rack mounted piston, divides a cylinder into two parts, and they are connected with a valve which ensures that pump is completely empty on one side of the cylinder while the other side is refilled to keep the flow direction same. The problem for two-sided piston cylinder assembly is its limited operating range in terms of peak flow because of the decreased stroke volume (Frayne et al., 1992; Salsac et al., 2006).

**Figure 11:**
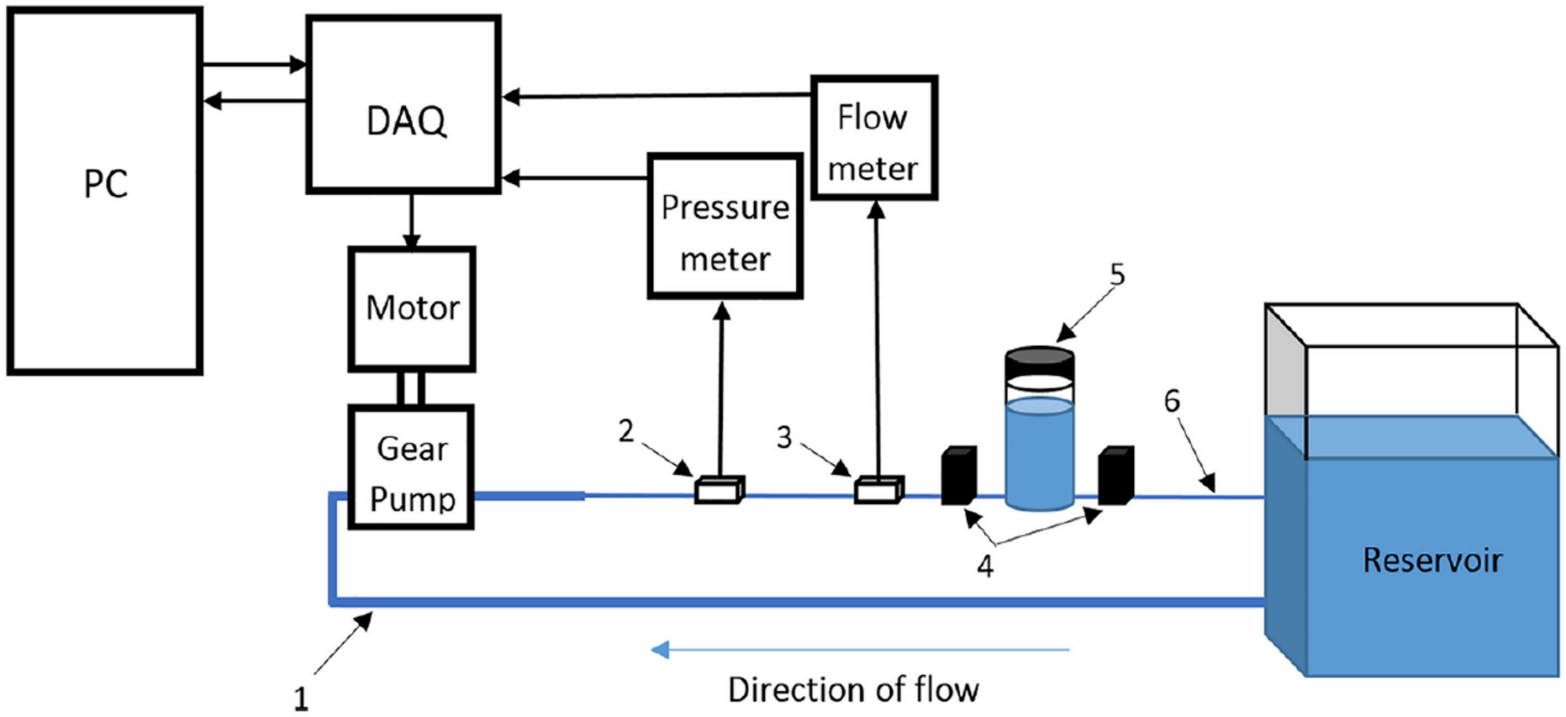
Schematic representation of flow circulatory system using computer-controlled gear pump with a feedback mechanism to achieve desired physiological flow and pressure waveform [The figure is adapted from Mechoor et al. (2016) and used with permission].

*Gear pumps.* In recent years, researchers have found that gear pumps are quite appropriate in physiological flow generation, where a controller needs to be integrated with a servo motor to run the gear pump. In that case, reverse flows can also be easily generated. An example of such system is presented in Figure 11 (Mechoor et al., 2016). Different from the aforementioned systems that generate pulsatility, computer-controlled gear pumps operate continuously without requiring any additional equipment and can replicate the physiological waveforms quite accurately (Gaillard and Deplano, 2005; Deplano et al., 2007, 2013, 2014; Mechoor et al., 2016). In addition, Mechoor et al. (2016) developed a closed loop system with integrating a feedback mechanism from output flow to the computer controlled gear pump system to better match the desired physiological flows and pressure waveforms.

##### Commercially available pumps

Besides these component-based in-house built systems, there are commercially available physiological flow generators. Harvard Pump (Harvard Apparatus, Holliston, MA) and SuperPump (Vivitro Labs, Victoria, BC, Canada) are commercially available pumps that are partially programmable and offer several number of arterial waveforms. A fully programmable commercial pump is CardioFlow (Shelley Medical Imaging Technologies, London, ON, Canada), which is again a two-sided piston pump that generates pulsatile flow, by dividing the cylinder into two chambers, each with fluid ports at the end. Some of the studies that utilize these pumps in hemodynamic analysis of different arteries are Pahlevan and Gharib (2013), Groves et al. (2014), and Najjari and Plesniak (2016). Resulting waveforms for these commercial pumps are very similar to physiological cases of predefined arteries, but the range of arteries that they can replicate is limited.

#### Piping and Pressure Compliance

As a component of flow circulatory systems, piping is critical in terms of obtaining correct inlet waveforms. The main concerns in piping are the general arrangement and the distance between the pump exit and test section inlet, which determine whether the fully developed flow is generated at the inlet of the test section. Some researchers specify this required distance as 100 times the diameter of the tubing before flow enters the test section (Moore et al., 1992). In a latter study, Durst et al. (2005) have suggested a formula, as given in Equation (17).

(17) $$\begin{array}{l} \frac{L}{D}={\left[0.619^{1.6}+{\left(0.0567Re\right)}^{1.6}\right]}^{1/1.6} \end{array}$$

where D is the tube diameter (m), L is the entrance length (m) and *Re* is the Reynolds number. Tsai and Savaş (2010) states in the study of cerebral saccular aneurysms that the entrance length required for fully developed flow as given in Equation (18).

(18) $$\begin{array}{l} L=0.058dRe \end{array}$$

He and Ku (1994) have claimed that the maximum length of the inlet pipe to satisfy the requirement of fully developed flow conditions for pulsatile flow is smaller than the tube length for steady flow conditions.

In order to match the pressure waveform at the inlet of the test section, various methods have been implemented. Researchers frequently employing downstream vascular simulators, replicate the impedances along the arteries to generate pressure waveform at specific arteries, which are generally composed of flow resistances and compliance chambers. In some recent studies, resistor–capacitor–resistor (RCR) module is adapted, which is composed of proximal and distal resistors (pinch valves) with a compliance chamber (air chamber) (Deplano et al., 2013; Mechoor et al., 2016). Besides all of these, reproducing physiological pressures throughout the aneurysm sac can be accomplished very accurately by the lumped-parameter boundary condition module, which is suggested by Kung et al. (2011). Such module is adapted downstream of aneurysm model to replicate outlet boundary condition, consisting of an inductance (L), proximal resistance (Rp), capacitance (C), and distal resistance (Rd), which is, in total, called as four-element Windkessel model.

#### Test Section

Another component of a circulatory flow setup is the model of the artery of interest, or so called, anatomical phantom. In early studies, an aneurysm model, which has transparent rigid walls with simplified geometry, has been integrated to flow loop (Egelhoff et al., 1999). These simplified and rigid models cannot replicate the exact compliant nature of the vessels. However, they provide insight about the governing physical processes occurring inside the AAA, including but not limited to separation of the flow at the inlet of AAA, vortex generation and jet regions, transition to turbulence, oscillatory flow structure, and high or low wall shear stress regions. With such controllable geometries, the parametric studies regarding aspect ratio, bulge diameter, and bulge asymmetry can be characterized. In recent investigations, more realistic geometries have been implemented. Rigid aneurysm models with simplified geometries are in the form of straight tube with a concentric bulge, which can be described by an ellipse formula (Yu, 2000), and are generally manufactured from glass (Salsac et al., 2006), or resins (Biglino et al., 2013). On the other hand, in order to understand exact hemodynamics and biomechanical forces that are generated through the aneurysms, it is necessary to work with compliant arterial models (Deplano et al. (2007) to replicate *in vivo* condition by manufacturing patient-specific phantom.

Manufacturing the realistic and patient specific arterial model is quite challenging. Different techniques are applied in literature by using different materials like silicone, polyurethane, and latex, where in the most well-known technique polyjet printing is utilized to manufacture transparent, 3D, and compliant phantoms (Sulaiman et al., 2008; Biglino et al., 2013; Ionita et al., 2014). 3D patient-specific vascular imaging data generated by means of CT scanner or MR techniques are collected and exported into Standard Tessellation Language (STL) file format, and this STL file is imported into 3D polyjet printer. The printer head generates layers of liquid photopolymer and builds them up to create 3D models. This technique enables to utilize different materials with a high resolution. As another method 3D printed vessel lumens are used as molds of the real phantoms that are going to be casted (Ho et al., 2017). For non-transparent phantoms, other techniques with non-transparent materials are also available to manufacture patient specific artery models where Doppler Ultrasound measurements are conducted for flow quantification. As an example, embedding silicone-elastomer vessel model into the agar based tissue-mimicking material (TMM) having same acoustic properties with artery (Poepping et al., 2004), direct machining of phantom using numerically controlled milling rather than producing a mold (Wong et al., 2008) or some other techniques (Watts et al., 2007; Allard et al., 2013) can be given. Even though Doppler Ultrasound technique does not require transparent phantoms, which may be an advantage, it provides only the maximum velocity in a flow cross section. Therefore, detailed hemodynamics evaluation is not possible with Doppler Ultrasound technique.

#### Blood-Mimicking Fluid

Blood is a non-Newtonian fluid, where the viscosity changes with shear rate. In literature, most researchers have utilized Newtonian fluids as blood-mimicking fluid because blood behaves like a Newtonian fluid for certain shear rate values (Berger and Jou, 2000). However, its non-Newtonian behavior becomes critical when shear rate is low and Deplano et al. (2013) reports that wall shear rate in some regions of AAA drops to values of 1 *s*^−1^. In this case, the blood acts as a non-Newtonian shear thinning fluid because viscosity decreases with increasing shear rate (Mandal, 2005). Non-Newtonian behavior of blood can be modeled using Carreau-Yasuda viscosity model, which is given in Equation (14). Most of the experimental studies utilize the Newtonian assumption, and generally pure water or the mixture of glycerin and water with different volumetric ratios have been widely used as blood mimicking fluid (Boutsianis et al., 2008; Stamatopoulos et al., 2010, 2011). There is only few studies that use non-Newtonian fluids of which behavior are very similar to the blood, like Xanthane Gum solution with glycerin (Deplano et al., 2014; Najjari and Plesniak, 2016).

### Flow Measurement System

In early studies, flow field is generally observed with the help of flow visualization technique (Budwig, 1994; Moore and Ku, 1994). The growing trend, which is the Particle Image Velocimetry (PIV) has been developed since the early 1980's to acquire velocity vector information of a whole flow field instantaneously with a high resolution (Adrian, 1991). Since 1993, PIV has been utilized in biologically important flows because of being a non-intrusive approach. In terms of the flow field information, different versions of PIV are available including 2D (planar) PIV, Stereoscopic PIV, Holographic PIV, and Tomographic PIV. In most of the AAA studies, planar PIV has been implemented, while Stereoscopic PIV has been utilized in a very few studies. As of authors knowledge, although Tomographic PIV has been applied to disturbed hemodynamics through intracranial aneurysms (Roloff et al., 2017, 2018), Holographic PIV and Tomographic PIV have not been applied to AAA studies yet.

Planar PIV is the mapping of average displacements of seeding particles within interrogation areas over a small time interval by means of two successive images of an illuminated plane in a fluid flow. Interrogation areas are small sub areas of each image, containing sufficient number of illuminated seeding particles. To find the displacements of particles for a short time interval, interrogation areas of two successive images are compared by means of cross correlation technique, resulting in determination of the most probable velocity vector for the particles inside of that interrogation areas, and this procedure continues until all interrogation areas are correlated and whole flow field velocity vectors are generated.

The fluid is seeded with neutrally buoyant particles, which should be sufficient in amount and size, typically varying from 5 to 50 μm, depending on the flow rate, magnification of the camera, and the field of view as well (Stamhuis, 2006). There are several types of seeding particles with respect to illumination characteristics, such as reflective, scattering, and fluorescent. Among all of these options, fluorescent particles have an advantage that fluorescent light can be distinguished from the illumination, which increases the visibility of the particles (Tsai and Savaş, 2010). Illumination of seeding particles with laser sheets and capturing the images with camera bring the refraction problem where the refractive indices of mediums and inclination angles of surfaces when lights passes through them are quite critical. Since the AAA models are curved in shape, in addition to the efforts given to match the refractive indices of working fluid and phantom, a box with flat surfaces perpendicular to the surface normal of camera lens plane covers the test section and is filled with working fluid to minimize refraction problems (Budwig, 1994).

**Figure 12:**
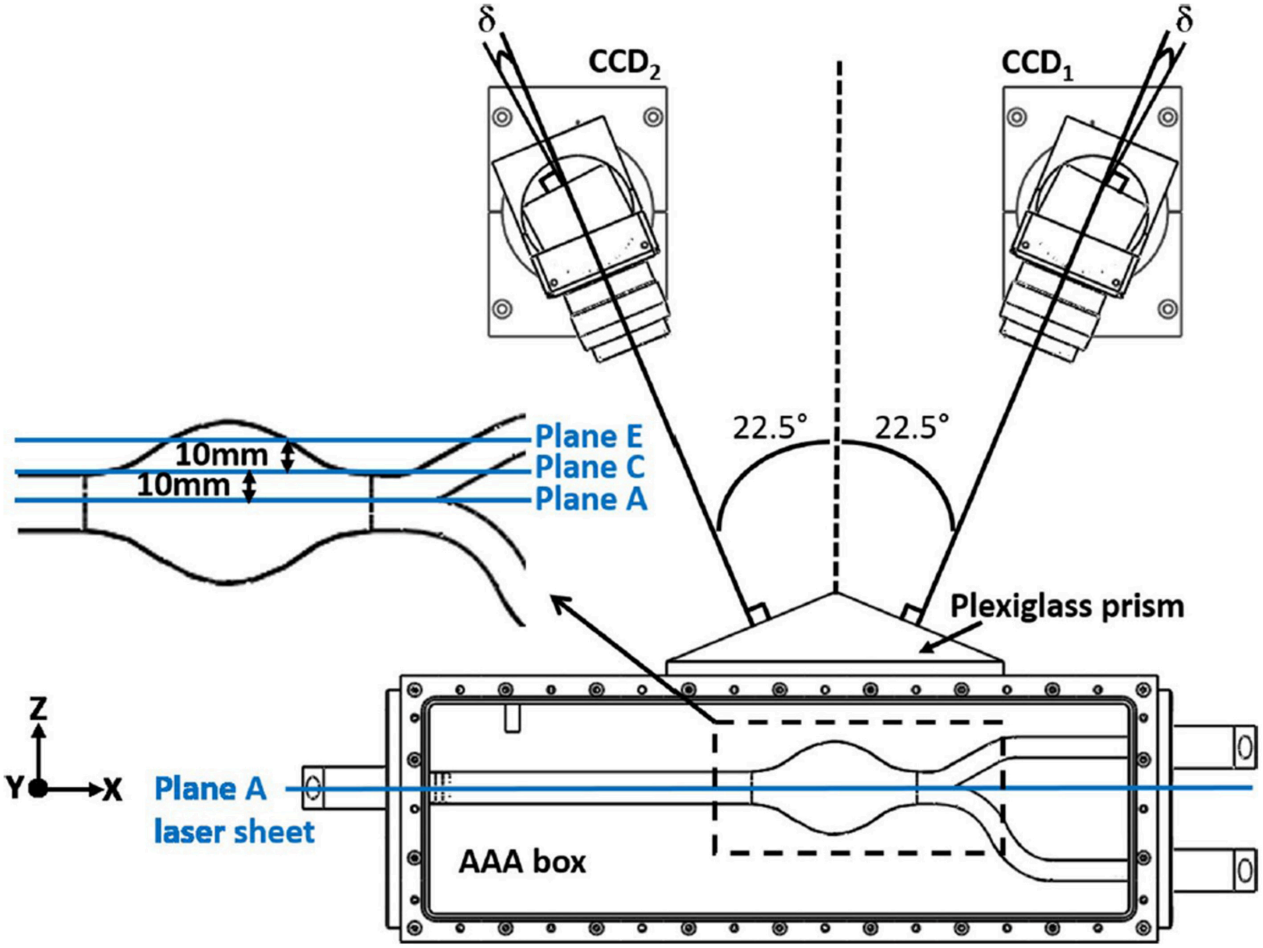
Typical camera arrangement of the SPIV. δ is the angle between the lens plane and the image plane, and faces of plexiglass prism is perpendicular to corresponding camera, which is used to minimize refraction related problems [The figure is adapted from Deplano et al. (2016) and used with permission].

In planar PIV, two component velocity information on 2-D plane is obtained using two laser sheets and an advanced camera, where in Stereoscopic PIV three-velocity components are obtained on 2-D plane using two cameras, as can be seen in Figure 12. Note that, lens planes and image planes of cameras are tilted to satisfy the Scheimpflug arrangement and plexiglass prisms in addition to the box for the test section are used to minimize refraction problems. In literature, there are many studies that utilize PIV technique to study the hemodynamics through AAA. In Table 1, PIV studies that have been conducted for the last 20 years are listed, considering the flow conditions at the inlet, type of blood mimicking fluids, wall types, geometry, and Reynolds number ranges.

**Table 1:** PIV studies conducted in the field of hemodynamics in AAA for the last 20 years.

**References**	**Flow generator**	**Blood mimicking fluid**	**Wall type**	**Geometry**	**Reynolds numbers**
Yu, 2000	Gravity driven pump with piston cylinder arrangement (Unsteady-Sinusoidal)	Newtonian fluid: A solution mixture of glycerin and water	Rigid wall-Pyrex glass tubes	Straight Tube and Axisymmetric, elliptical shaped bulge	Steady Flow: 400–1400 Unsteady Flow, Peak Value: 1,274
Salsac et al., 2006	Two sided piston cylinder arrangement (Unsteady-Physiological)	Newtonian fluid: Pure water	Rigid wall-Glass	Axisymmetric bulges with different diameters	Unsteady flow, Peak Value: 2,700
Deplano et al., 2007	Computer controlled pump (Unsteady-Physiological)	Newtonian fluid: Aqueous glycerin solution, 60% water	Rigid wall-Glass Compliant wall-Molded polyurethane	Asymmetric bulge	–
Boutsianis et al., 2008	Variable speed gear pump (Steady)	Newtonian fluid: A mixture of 40% water, 60% glycerol	Compliant wall-Silicone phantom	Patient-specific aneurysm, gathered by CT scanning	Steady flow: 560
Stamatopoulos et al., 2010	Linear reciprocating piston cylinder arrangement (Unsteady-Sinusoidal)	Newtonian fluid: A water and glycerin solution (40:60 by volume)	Rigid wall-Elastomer material (Sylgard-184)	Axisymmetric, elliptical shaped bulge	Steady Flow: 105–690 Unsteady Flow, 105–690
Stamatopoulos et al., 2011	Linear reciprocating piston cylinder arrangement (Unsteady-Physiological)	Newtonian fluid: A water and glycerin solution (40:60 by volume)	Compliant wall-Liquid silicon elastomer	Patient-specific aneurysm, gathered by CT scanning	Unsteady flow, Peak Value: 541
Deplano et al., 2013	Computer controlled pump (Unsteady-Physiological)	Newtonian fluid: Aqueous glycerin solution, 60% water	Compliant wall-Molded polyurethane	Asymmetric bulge with symmetric and asymmetric iliac bifurcation	Unsteady Flow, Peak Value: 1,876
Chen et al., 2014	Steady Submersible Pump	Newtonian fluid: A mixture of NaI and water	Rigid wall	Patient-specific aneurysm, produced by rapid prototyping	Steady Flow: 2,234
Deplano et al., 2014	Computer controlled pump (Unsteady-Physiological)	Shear-thinning fluid: Aqueous solution of Xanthane gum (XG)	Compliant wall-Molded polyurethane	Asymmetric bulge with asymmetric iliac bifurcation	Unsteady Flow, Peak Value: 1,941
Deplano et al., 2016	Computer controlled pump (Unsteady-Physiological)	Shear-thinning fluid: Aqueous solution of Xanthane gum (XG)	Compliant wall-Molded polyurethane	Asymmetric bulge with asymmetric iliac bifurcation	Unsteady Flow, Peak Value: 2,298
Wang et al., 2016	Piston cylinder arrangement (Unsteady-Physiological)	Newtonian fluid: Pure water	Rigid wall-Glass	Straight Tube and Axisymmetric, elliptical shaped bulge	

### Scaling Parameters

Pulsatile flow through the arteries is generally characterized by two non-dimensional parameters, Reynolds and Womersley numbers, which are defined as the Equations (19,20).

(19) $$\begin{array}{l} Re=\;\frac{UD}{\nu } \end{array}$$

(20) $$\begin{array}{l} \alpha =0.5D\sqrt{\frac{\omega }{\nu }} \end{array}$$

where *U* is the velocity, D is the tube diameter, ν is the kinematic viscosity and ω is the frequency of periodicity (Womersley, 1955). Reynolds number is the ratio of inertia forces to viscous forces, which determines the flow regime and varies from 1 for very small arteries to ~4,000 for aorta. Womersley number is the ratio of unsteady inertia forces to viscous forces, which can also be represented in terms of Reynolds number and Strouhal number. For low Womersley number flows, viscous forces dominate and velocity profile inside the arteries become parabolic, and velocity at the centerline oscillates with a phase of driving pressure gradient. For high Womersley number flows (α > 10), unsteady inertia forces dominate and velocity profile has a nearly flat velocity profile, and in that case, flow represents situations with rapid acceleration and deceleration (Womersley, 1955; Ku, 1997). Womersley number value for femoral artery is nearly four, while for aorta, it reaches up to 22.

These non-dimensional parameters are required to be kept constant when scaling is conducted, enabling to perform similarity analysis. The artery models that are studied during the experiments might be scaled to a larger size because generally the vessels in human circulatory system are relatively small for macro scale investigations. In that case, the normal vessel is the prototype with exact dimensions and velocity values, while the artery model utilized in experiments might be the scaled version of the prototype. In that case, performing a similarity analysis by matching Reynolds and Womersley numbers of prototype and model will mimic physiological conditions and enables the simulation of performance of the prototype. By equating the Reynolds and Womersley numbers for the model and prototype, as given in Equations (21,22).

(21) $$\begin{array}{l} Re_{m}={\;Re}_{p},\;\frac{U_{m}D_{m}}{\nu_{m}}=\frac{U_{p}D_{p}}{\nu_{p}} \end{array}$$

(22) $$\begin{array}{l} \alpha_{m}=\alpha_{p},\;0.5D_{m}\sqrt{\frac{\omega_{m}}{\nu_{m}}}=0.5D_{p}\sqrt{\frac{\omega_{p}}{\nu_{p}}} \end{array}$$

where subscript m is representing model and p is representing prototype and with the following ratio of diameters of arteries and kinematic viscosities, which is given in Equation (23).

(23) $$\begin{array}{l} L_{r}=\frac{D_{p}}{D_{m}}=constant,{\;\nu }_{r}=\frac{\nu_{p}}{\nu_{m}}=constant \end{array}$$

where *L*_*r*_ is diameter ratio and ν_*r*_ is the kinematic viscosity ratio of prototype to model, the velocity and frequency of the model become

(24) $$\begin{array}{l} U_{m}=\frac{{U_{p}L}_{r}}{\nu_{r}},\;\omega_{m}=\;\frac{{\omega_{p}L}_{r}^{2}}{\nu_{r}} \end{array}$$

To increase the resolution of a small vessel by increasing its diameter, the velocity should be decreased by the ratio of 1/*L*_*r*_ to match the Reynolds number, while frequency should be decreased by the ratio of ${1/L}_{r}^{2}$ to match the Womersley number. In certain test setups, the frequency adjustment might be needed possibly due to the following two reasons: (1) Flow circulatory system could not reach the desired frequencies for the flow waveform, (2) Flow measurement system could not provide enough sample in one cycle. In that case, the desired velocity and diameter for the setup are determined accordingly. Similarly, if the flow rate in the setup is the limitation, then once the velocity is determined, diameter of the setup is adjusted according to the Reynolds number and finally the frequency of the waveform is set based on the Womersley number to ultimately reach the similarity.

### Key Parameters to Study in Experimental Investigation of Hemodynamics of AAA

In experimental studies, researchers have generally reported the results of the hemodynamics in AAA in terms of velocity and vorticity parameters (Salsac et al., 2006; Stamatopoulos et al., 2010) obtained using several vortex identification techniques, such as swirling strength (Deplano et al., 2007). Swirling strength of a vortex is defined by λ_*ci*_, while ${\lambda_{ci}}^{2}$ is called enstrophy, which is energy of vorticity and utilized for decreasing background noise (Zhou et al., 1999).

In recent years, with the utilization of Stereoscopic PIV technique, some researchers have also presented the 3D vortex evaluation through aneurysm sac (Deplano et al., 2016). In this technique, Deplano et al. (2016) have focused on the effect of transverse velocity component (*w*) measurement by means of comparing its magnitude to other velocity components, such as axial (*u*), vertical (*v*), or total velocity vector (**V**), of which magnitude is

(25) $$\begin{array}{l} ||\text{V}||\;=\sqrt{u^{2}+v^{2}+w^{2}} \end{array}$$

In addition, the magnitude of 2D velocity vector, (**V**_**2D**_), can also be written as

(26) $$\begin{array}{l} ||{\text{V}}_{2\text{D}}||\;=\sqrt{u^{2}+v^{2}} \end{array}$$

For each spatial point of different planes inside aneurysm, relative difference, *RD* between these two velocity magnitudes is calculated as

(27) $$\begin{array}{l} RD=\;\frac{||\text{V}||-||{\text{V}}_{2\text{D}}||\;}{||\text{V}||\;}\times 100 \end{array}$$

Once the *RD* values are calculated at each point, the mean value of the relative difference, *RD*, can be obtained on the corresponding planes. If *RD*, is lower than 10%, it can be interpreted as *u* and *v* components are sufficient to represent flow behavior, whereas for higher mean relative difference values, *w* component needs to be included (Deplano et al., 2016). The quantities such as $\frac{|w|\;}{||\text{V}||\;}$ contours and $\frac{|w|\;}{|u|\;}$, $\frac{|w|\;}{|v|\;}$, *RD* values, which underline the specific importance of *w* with respect to *u* and *v* components, can be utilized in order to investigate the importance of transverse velocity component (*w*) on hemodynamics and AAA progression. In addition, vortex ring and its strain dynamics which can also be obtained from Stereoscopic PIV data, are used to understand different flow instabilities and growth mechanism (Deplano et al., 2016). The rate of deformation tensor can be decomposed into rate of strain tensor, which is pure rate of deformation tensor *S*_*ij*_, and pure rate of rotation tensor Ω_*ij*_. The strain rate tensor S enables to visualize and quantify the direction of compression and stretching. If the eigenvalues $S_{ij}^{*}$ of tensor S are positive, the fluid element is stretched while they are negative, the element is compressed (Bouremel et al., 2009).

### Important Findings on Hemodynamics of AAAs From Experimental Studies

#### Hemodynamics in Undilated Aorta

In early studies, researchers have focused on hemodynamics inside undilated aorta to understand the effect of flow on dilation of arterial wall. Ku et al. (1989) investigated undilated abdominal aorta by using a realistic glass abdominal aorta model and observed steady flow inside that artery by injecting a dye into the model. Flow pattern at the infrarenal aorta, where AAA generation is observed, has more complex structure than in the suprarenal aorta because of the following reasons: (1) The aorta has a curvature at that section, which localizes transient separation to its posterior wall; (2) There are branches of arteries transferring blood to kidneys after the suprarenal and before the infrarenal segments. The blood flow transferred from these branches creates secondary flows at the upstream of infrarenal aorta; (3) The bifurcation at the downstream of infrarenal aorta is creating a horseshoe vortex. All these unsteady flow structures make the infrarenal segment appropriate to generate an aneurysmal bulge.

#### Hemodynamics in AAA

##### Effect of pulsatility

In early studies on AAA (Nerem, 1984; Egelhoff et al., 1999; Yu, 2000), steady flow analysis has been conducted and as reported under laminar conditions, a jet of fluid passing though the core of the aneurysm is surrounded by a circulating vortex. The flow velocity through the center of the aneurysm is 40 times higher than the velocity at the recirculation region, and WSS value has the largest peak at the reattachment point of this recirculating flow. The WSS magnitude in the recirculation region is about ten times less than the value in the upstream tube, because the core flow separation at the bulge causes the negative WSS values. Scherer (1973) reports that the transition to turbulence begins at Re = 2900. Asbury et al. (1995) studied AAA without any bifurcation and utilized Color Doppler Flow Imaging (CDFI) method to visualize the flow and Laser Doppler Velocimetry (LDV) method to quantify the flow in order to address the effect of bulge diameter and turbulence on hemodynamics. They reported a correlation between the aneurysm size and rupture because especially in larger aneurysms turbulent flow is observed, which causes higher WSS values throughout the bulge (Asbury et al., 1995).

The cycle of physiological flow with strong acceleration and deceleration of flow in AAA has strong effects on vortex dynamics, which moves from proximal to the distal end creating significant fluctuations in WSS and pressure though the aneurysm bulge. Fukushima et al. (1989) conducted the first experimental and numerical study on pulsatile flow conditions for glass models of axisymmetric and asymmetric AAAs. They observed that primary and secondary vortices were present inside the bulge, and reported that the peak negative shear stress occurs at the distal end. Yu (2000) performed the first PIV experiments for both steady and pulsatile flow waveforms in pyrex glass aneurysm models without any iliac bifurcation to see the effects on sinusoidal waveform on hemodynamics. Yu and Zhao (2000) compared the steady and pulsatile flow dynamics in a straight tube with a side bulge using PIV, and they reported significant differences between flow patterns. Stamatopoulos et al. (2010) reported the difference between steady and pulsatile, sinusoidal flow in AAA, where the flow separation at the proximal end and reattachment at the distal end of aneurysm bulge is witnessed for steady case, but for unsteady case, these locations vary for each instant of cardiac cycle. However, the peak WSS values are observed at the model exit for both flow conditions.

##### Effects of wall material properties, working fluid properties, and geometric parameters on AAA hemodynamics

**Figure 13:**
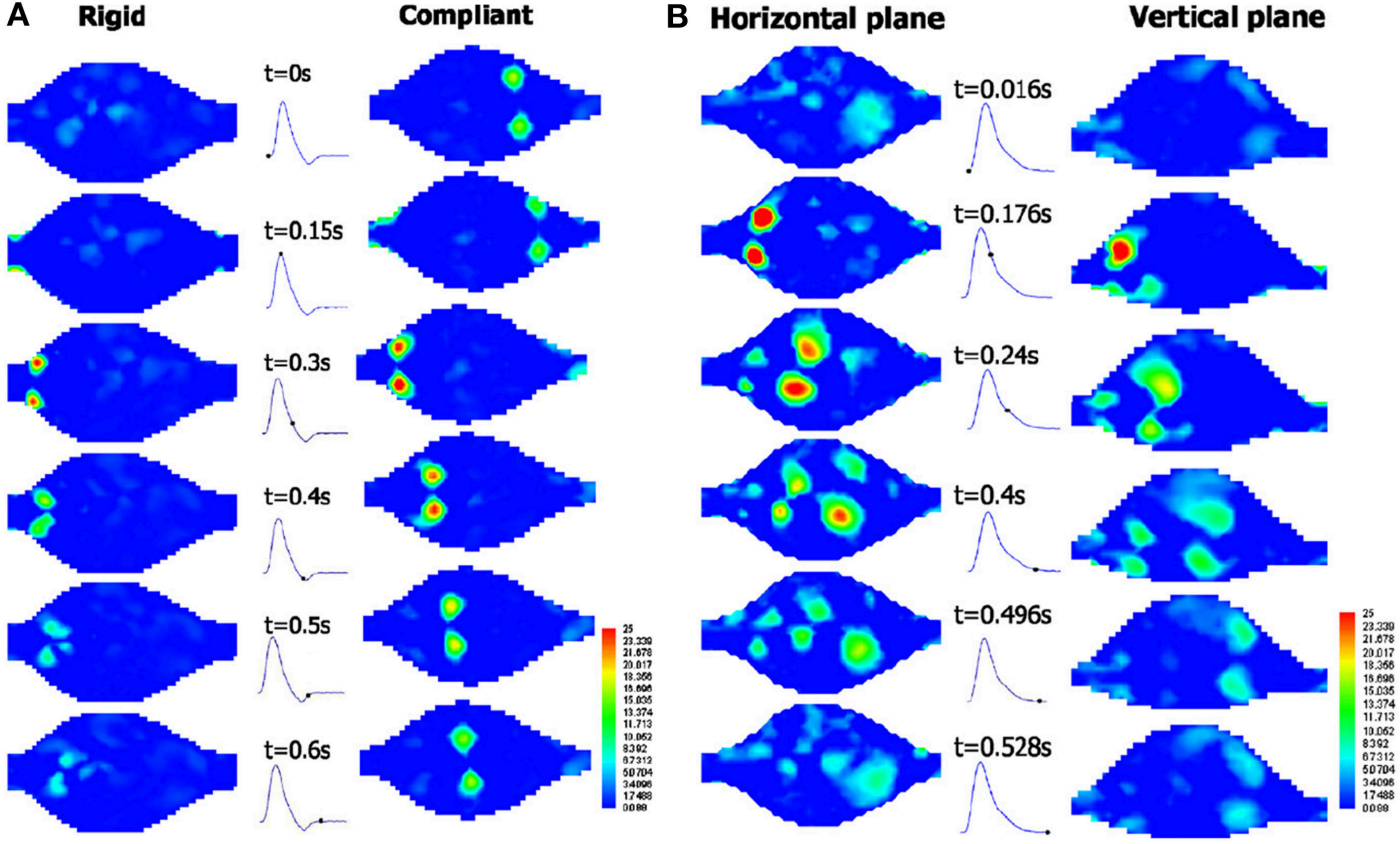
Temporal evolution of the swirling strength λ_*ci*_ at different time instants in **(A)** the rigid model and the compliant model in horizontal plane, and **(B)** the compliant model at horizontal and vertical planes, under exercise conditions. During the deceleration phase for the compliant model, vortices impact on the walls and swirling strength increases, including vortex shedding occurrence [The figure is adapted from Deplano et al. (2007) and used with permission].

In most of the experimental studies, rigid axisymmetric models were used to investigate the hemodynamics in aneurysm bulge (Yu, 2000; Salsac et al., 2006; Stamatopoulos et al., 2010). Deplano et al. (2007) and Meyer et al. (2011) have tested two aneurysm models one having rigid and the other with compliant walls. The rigid aneurysm was made of glass while the compliant one was made of molded polyurethane, and both had the identical geometry as a simple bulge for comparison purposes. As can be seen in Figure 13, there is significant difference between the flow fields inside both of the aneurysms. In rigid model, there is only viscous dissipation, while in compliant model, viscoelastic dissipation is also observed, which is indicated as more critical. During the accelerating phase of the cycle, compliant walls absorb kinetic energy in the potential energy form, leading the walls become expanded. While the flow is decelerating, this stored kinetic energy retracts the walls. This expansion and retraction contribute the progression of vortices at the distal end of aneurysm during the deceleration phase. They conclude that increasing wall compliance causes collision of vortices with the walls, increasing both the local pressures and wall stresses especially at the distal end (Deplano et al., 2007; Meyer et al., 2011). Producing models with exact *in vivo* wall material properties and thicknesses are also important to decide rupture location. In literature, there are several studies focusing on identifying rupture location in compliant AAA models by means of tensile tests (Doyle et al., 2009a, 2010) and biaxial tests (O'Leary et al., 2014). Mechanical behaviors of ILT and wall calcification and their effects on rupture have also been characterized by means of these tests (Ene et al., 2011; O'Leary et al., 2013, 2015).

**Figure 14:**
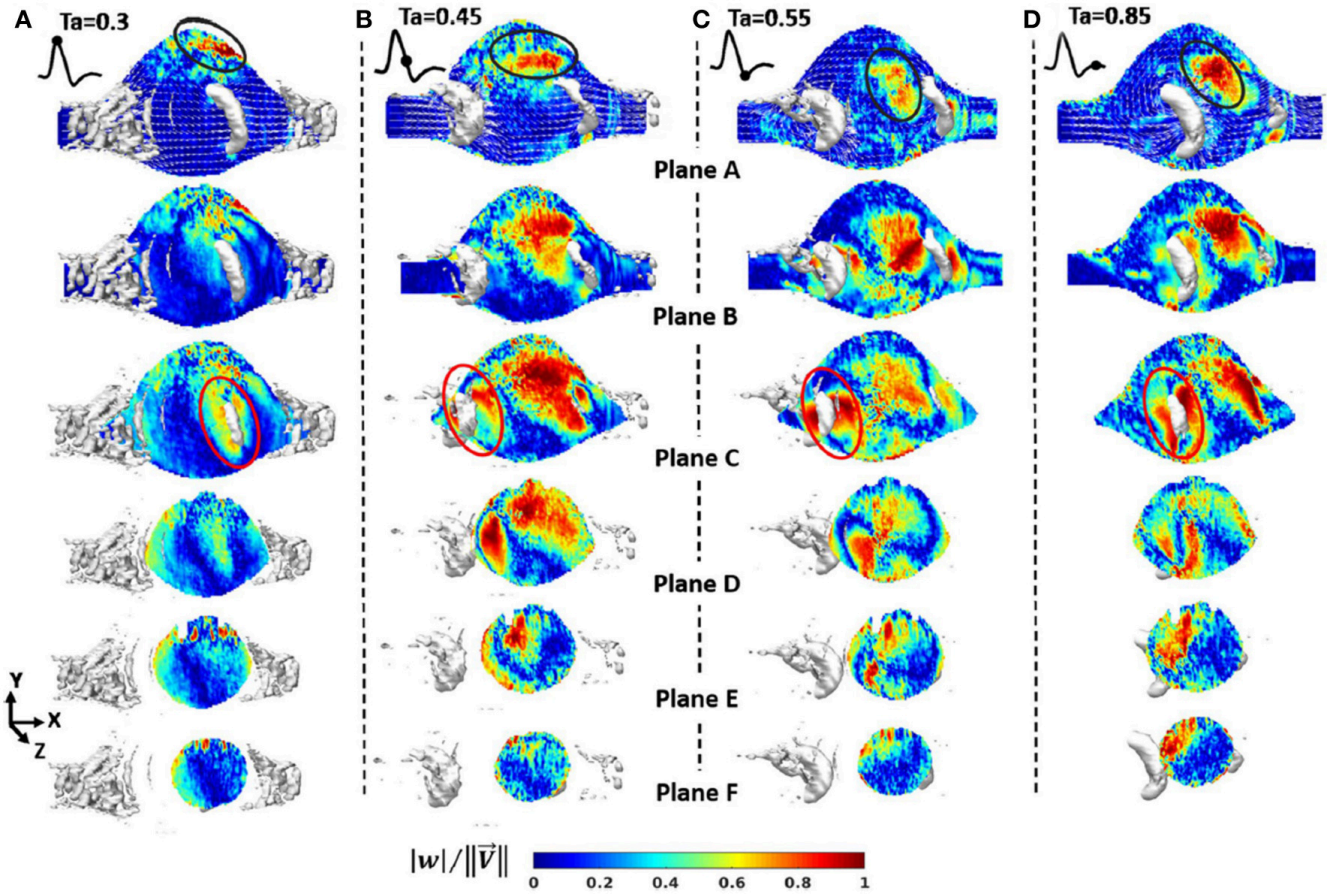
Contours of $\frac{|w|\;}{||V||\;}\;$from Stereoscopic PIV measurements in six planes and four time instants of cardiac cycle **(A)** *T*_*a*_ = 0.3; maximum flow rate, **(B)** *T*_*a*_ = 0.45; decelerating flow rate, **(C)** *T*_*a*_ = 0.55; minimum flow rate, **(D)** *T*_*a*_ = 0.85; flow rate is nearly zero. Velocity vector projection and isosurface λ_*ci*_ = 8 (in white) is imposed on each plane. Flow stagnation area (in black) is represented by weak components of velocity vectors [The figure is adapted from Deplano et al. (2016) and used with permission].

The real aneurysms also contain iliac bifurcation. Deplano et al. (2013) have studied asymmetric aorto-iliac bifurcation for a simple bulge. Two artery models, one with a straight outlet tube and the other with iliac bifurcation have been studied to observe the effect of bifurcation on flow structure. The results indicate that for the model with iliac bifurcations, intensity of the vortex ring impact on the anterior wall is about 90% higher than without bifurcation. When the vortex ring impinges on the wall, the forces generated by this impingement might possibly cause the rupture (Chu et al., 1995). For a compliant aneurysm bulge with aorta iliac bifurcation, stereoscopic PIV technique has been implemented to observe vortex ring impingement inside the aneurysm as 3D quantification (Deplano et al., 2016). In Figure 14, contours of $\frac{|w|\;}{||\text{V}||\;}$ in six planes and four time instants of cardiac cycle is represented in order to understand the effect of transverse velocity component, *w*. During the deceleration phase, where *T*_*a*_ = 0.45, 0.55 *and* 0.85, the magnitude of the mean transverse velocity component, *w*, is 0.7 times the axial component and 1.7 times the vertical component, especially for C and D planes which are located within the AAA bulge.

The other important concern about AAA is intraluminal thrombus (ILT) developed inside the aneurysms, consisting of blood proteins, platelets, and cells. Platelets stick the vortex ring generated inside the aneurysm throughout the cardiac cycle, and are transported from proximal to distal end, released during vortex break up. They adhere to low wall shear stress sites, which will lead to thrombus formation (Biasetti et al., 2011; O'Rourke et al., 2012). The question that ILT formation decreases the rupture risk or not still has controversy. In an experimental and numerical study on AAA with and without ILT, it is reported that a recirculation region is generated inside AAA without thrombus while it is disappeared in AAA with ILT (Chen et al., 2014), and there are several studies that are seeking the governing hemodynamic parameters affecting platelet deposition (Deplano et al., 2013, 2014).

As the patient-specific studies, there are a few examples such as Particle Tracking Velocimetry (PTV) study of Boutsianis et al. (2008) and PIV study of Stamatopoulos et al. (2011), where they report that WSS have the peak values at the proximal and distal sites, like in simplified bulges, and the velocity waveform at the anterior site is highly disturbed because the vortex is generated and transmitted to downstream at this site, rather than posterior.

For mimicking the shear stress and shear strain rate relation of blood, Deplano et al. (2014) have used Xanthane Gum dissolved in aqueous solutions of glycerol, a shear thinning fluid, where they report that shear thinning fluid model imposes higher shear stress values throughout the aneurysm bulge than Newtonian model. They conclude that the rheology of working fluid affects the hemodynamics inside aneurysm model, and should be taken into account for more realistic experimentation. On the other hand, in another study which is performed on a curved artery model with again a shear thinning fluid, mixture of fluids like Xanthane Gum, glycerin, water, and sodium iodide (Najjari and Plesniak, 2016). The results are very similar to the studies for curved arteries with Newtonian fluids, concluding that for large arteries with large flow rates, rheology of the blood does not affect the flow structure. However, at that point it is important to note that, they have utilized comparatively high sodium iodide concentration in their mixture than required to resolve the refractive index problems, leading that their working fluid is less viscoelastic than the blood.

## Conclusion

Experimental and computational studies indicate that a certain number of parameters can be interpreted as the indicators of the rupture. Typically, the peak values of WSS and wall stress (structural stress) are interpreted as the footprints of an upcoming rupture. Some key parameters that are derived from WSS, such as TAWSS, OSI, and ECAP are also utilized to detect candidate locations of the rupture (Arzani and Shadden, 2015; Arzani, 2018; Singh et al., 2018). Unfortunately, determining the exact location of rupture by deciding the most effective hemodynamic parameter is still a controversial issue. For improved rupture risk assessment, ILT and plaque formations, patient-specific boundary conditions, arterial thickness variation, and heterogeneous material properties of arterial wall should be considered in a comprehensive computational analysis. For the experimental studies, flow loop should exactly replicate the *in-vivo* characteristics using a programmable, real time physiological pump that can supply re-producible and realistic inflow conditions to test model (Mechoor et al., 2016). In addition, lumped-parameter outlet boundary condition modules should be used to produce physiological pressures together with a compliant artery model having a patient specific geometry. The main purpose of these studies, in the end, should be to obtain a clinically useful tool in decision making step to understand whether the repair is necessary or not. In addition to mechanical analyses, the genetic aspect of the problem should be taken into account, since the disturbed biomechanical environment inside the aneurysm may affect gene expression patterns which may result in altered growth at later stages (Krishna et al., 2010).

## Author Contributions

HS and BR wrote the first draft and revised the manuscript. MY and HY structured, reviewed, and revised the manuscript. All authors read and approved the submitted version of the manuscript.

### Conflict of Interest Statement

The authors declare that the research was conducted in the absence of any commercial or financial relationships that could be construed as a potential conflict of interest.

## References

[B1] Adolph R. Vorp D. A. Steed D. L. Webster M. W. Kameneva M. V. Watkins S. C. (1997). Cellular content and permeability of intraluminal thrombus in abdominal aortic aneurysm. J. Vasc. Surg. 25, 916–926. 10.1016/S0741-5214(97)70223-4 9152321

[B2] Adrian R. J. (1991). Particle-imaging Techniques for experimental fluid mechanics. Annu. Rev. Fluid Mech. 23, 261–304. 10.1146/annurev.fl.23.010191.001401

[B3] Allard L. Soulez G. Chayer B. Qin Z. Roy D. Cloutier G. (2013). A multimodality vascular imaging phantom of an abdominal aortic aneurysm with a visible thrombus. Med. Phys. 40:063701. 10.1118/1.4803497 23718616

[B4] Amindari A. Saltik L. Kirkkopru K. Yacoub M. Yalcin H. C. (2017). Assessment of calcified aortic valve leaflet deformations and blood flow dynamics using fluid-structure interaction modeling. Inf. Med. Unlock. 9, 191–199. 10.1016/j.imu.2017.09.001

[B5] Anjum A. von Allmen R. Greenhalgh R. Powell J. T. (2012). Explaining the decrease in mortality from abdominal aortic aneurysm rupture. BJS 99, 637–645. 10.1002/bjs.8698 22473277

[B6] Arzani A. (2018). Accounting for residence-time in blood rheology models: do we really need non-Newtonian blood flow modelling in large arteries? J. R. Soc. Interface 15:20180486. 10.1098/rsif.2018.0486 30257924 PMC6170779

[B7] Arzani A. Gambaruto A. Chen G. Shadden S. (2017). Wall shear stress exposure time: a Lagrangian measure of near-wall stagnation and concentration in cardiovascular flows. Biomech. Model. Mechanobiol. 16, 787–803. 10.1007/s10237-016-0853-7 27858174

[B8] Arzani A. Shadden S. C. (2015). Characterizations and correlations of wall shear stress in aneurysmal flow. J. Biomech. Eng. 138:014503. 10.1115/1.4032056 26592536 PMC4844249

[B9] Arzani A. Suh G.-Y. Dalman R. L. Shadden S. C. (2014). A longitudinal comparison of hemodynamics and intraluminal thrombus deposition in abdominal aortic aneurysms. Am . J. Physiol. Heart Circ. Physiol. 307, H1786–H1795. 10.1152/ajpheart.00461.2014 25326533 PMC4269702

[B10] Asbury C. L. Ruberti J. W. Bluth E. I. Peattie R. A. (1995). Experimental investigation of steady flow in rigid models of abdominal aortic aneurysms. Ann. Biomed. Eng. 23, 29–39. 10.1007/BF02368298 7762880

[B11] Bathe K.-J. Hou Z. Ji S. (1999). Finite element analysis of fluid flows fully coupled with structural interactions. Comput. Struct. 72, 1–16. 10.1016/S0045-7949(99)00042-5

[B12] Bathe K.-J. Zhang H. (2004). Finite element developments for general fluid flows with structural interactions. Int. J. Numer. Methods Eng. 60, 213–232. 10.1002/nme.959

[B13] Bengtsson H. Bergqvist D. (1993). Ruptured abdominal aortic aneurysm: a population-based study. J. Vasc. Surg. 18, 74–80. 10.1067/mva.1993.42107 8326662

[B14] Berger S. A. Jou L. D. (2000). Flows in Stenotic Vessels. Annu. Rev. Fluid Mech. 32, 347–382. 10.1146/annurev.fluid.32.1.347

[B15] Biasetti J. Hussain F. Gasser T. C. (2011). Blood flow and coherent vortices in the normal and aneurysmatic aortas: a fluid dynamical approach to intra-luminal thrombus formation. J. R. Soc. Interface. 8, 1449–61. 10.1098/rsif.2011.0041 21471188 PMC3163425

[B16] Biasetti J. Spazzini P. G. Swedenborg J. Gasser T. (2012). An integrated fluid-chemical model toward modeling the formation of intra-luminal thrombus in abdominal aortic aneurysms. Front. Physiol. 3:266. 10.3389/fphys.2012.00266 22934022 PMC3429042

[B17] Biglino G. Verschueren P. Zegels R. Taylor A. M. Schievano S. (2013). Rapid prototyping compliant arterial phantoms for *in-vitro* studies and device testing. J. Cardiovasc. Magn. Reson. 15:2. 10.1186/1532-429X-15-2 23324211 PMC3564729

[B18] Bluestein D. Dumont K. De Beule M. Ricotta J. Impellizzeri P. Verhegghe B.. (2009). Intraluminal thrombus and risk of rupture in patient specific abdominal aortic aneurysm – FSI modelling. Comput. Methods Biomech. Biomed. Eng. 12, 73–81. 10.1080/10255840802176396 18651282

[B19] Bluestein D. Niu L. Schoephoerster R. T. Dewanjee M. K. (1996). Steady flow in an aneurysm model: correlation between fluid dynamics and blood platelet deposition. J. Biomech. Eng. 118, 280–286. 10.1115/1.2796008 8872248

[B20] Böckler D. Holden A. Thompson M. Hayes P. Krievins D. de Vries J.-P. P. M.. (2015). Multicenter nellix endovascular aneurysm sealing system experience in aneurysm sac sealing. J. Vasc. Surg. 62, 290–298. 10.1016/j.jvs.2015.03.031 25953017

[B21] Bouremel Y. Yianneskis M. Ducci A. (2009). On the utilisation of vorticity and strain dynamics for improved analysis of stirred processes. Chem. Eng. Res. Des. 87, 377–385. 10.1016/j.cherd.2008.11.016

[B22] Boutsianis E. Guala M. Olgac U. Wildermuth S. Hoyer K. Ventikos Y.. (2008). CFD and PTV steady flow investigation in an anatomically accurate abdominal aortic aneurysm. J. Biomech. Eng. 131:011008. 10.1115/1.3002886 19045924

[B23] Budwig R. (1994). Refractive index matching methods for liquid flow investigations. Exp. Fluids 17, 350–355. 10.1007/BF01874416

[B24] Canchi T. Saxena A. Ng E. Y. K. Pwee E. C. H. Narayanan S. (2018). Application of fluid–structure interaction methods to estimate the mechanics of rupture in asian abdominal aortic aneurysms. Bionanoscience 8, 1035–1044. 10.1007/s12668-018-0554-z

[B25] Casciaro M. E. Dottori J. El-Batti S. Alsac J.-M. Mousseaux E. Larrabide I.. (2018). Effects on aortoiliac fluid dynamics after endovascular sealing of abdominal aneurysms. Vasc. Endovascular Surg. 52, 621–628. 10.1177/1538574418791059 30058480

[B26] Celik I. Klein M. Janicka J. (2009). Assessment measures for engineering LES applications. J. Fluids Eng. 131:031102 10.1115/1.3059703

[B27] Chandra S. Raut S. S. Jana A. Biederman R. W. Doyle M. Muluk S. C.. (2013). Fluid-structure interaction modeling of abdominal aortic aneurysms: the impact of patient-specific inflow conditions and fluid/solid coupling. J. Biomech. Eng. 135, 081001. 10.1115/1.4024275 23719760 PMC3705803

[B28] Chen C.-Y. Antón R. Hung M.-Y. Menon P. Finol E. A. Pekkan K. (2014). Effects of intraluminal thrombus on patient-specific abdominal aortic aneurysm hemodynamics via stereoscopic particle image velocity and computational fluid dynamics modeling. J. Biomech. Eng. 136:031001. 10.1115/1.4026160 24316984 PMC5101028

[B29] Chen C. Y. Hung M. Y. Finol E. A. Pekkan K. (2015). Experimental and computational investigation of the patient-specific abdominal aortic aneurysm pressure field AU - Antón, R. Comput. Methods Biomech. Biomed. Eng. 18, 981–992. 10.1080/10255842.2013.865024 24460046

[B30] Choudhury S. Anupindi K. Patnaik B. S. V. (2019). Influence of wall shear stress and geometry on the lumen surface concentration of low density lipoprotein in a model abdominal aortic aneurysm. Phys. Fluids 31:011901 10.1063/1.5074125

[B31] Chu C. C. Wang C. T. Chang C. C. (1995). A vortex ring impinging on a solid plane surface—Vortex structure and surface force. Phys. Fluids 7, 1391–1401. 10.1063/1.868527

[B32] Corbett T. J. Doyle B. J. Callanan A. Walsh M. T. McGloughlin T. M. (2009). Engineering silicone rubbers for *in vitro* studies: creating AAA models and ILT analogues with physiological properties. J. Biomech. Eng. 132:011008. 10.1115/1.4000156 20524746 PMC2882675

[B33] Cosford P. A. Leng G. C. (2007). Screening for Abdominal Aortic Aneurysm. The Cochrane database of systematic reviews. Available online at: http://europepmc.org/abstract/MED/17443519 (accessed April 24, 2019) 10.1002/14651858.CD002945.pub2 17443519

[B34] Darling R. C. Messina C. R. Brewster D. C. Ottinger L. W. (1977). Autopsy study of unoperated abdominal aortic aneurysms. The case for early resection. Circulation 56 (3 Suppl.), II161–164. 884821

[B35] Deplano V. Guivier-Curien C. Bertrand E. (2016). 3D analysis of vortical structures in an abdominal aortic aneurysm by stereoscopic PIV. Exp. Fluids 57:167 10.1007/s00348-016-2263-0

[B36] Deplano V. Knapp Y. Bailly L. Bertrand E. (2014). Flow of a blood analogue fluid in a compliant abdominal aortic aneurysm model: experimental modelling. J. Biomech. 47, 1262–1269. 10.1016/j.jbiomech.2014.02.026 24612986

[B37] Deplano V. Knapp Y. Bertrand E. Gaillard E. (2007). Flow behaviour in an asymmetric compliant experimental model for abdominal aortic aneurysm. J. Biomech. 40, 2406–2413. 10.1016/j.jbiomech.2006.11.017 17258220

[B38] Deplano V. Meyer C. Guivier-Curien C. Bertrand E. (2013). New insights into the understanding of flow dynamics in an *in vitro* model for abdominal aortic aneurysms. Med. Eng. Phys. 35, 800–809. 10.1016/j.medengphy.2012.08.010 22981221

[B39] Di Achille P. Tellides G. Figueroa C. A. Humphrey J. D. (2014). A haemodynamic predictor of intraluminal thrombus formation in abdominal aortic aneurysms. Proc. R. Soc. A 470:20140163 10.1098/rspa.2014.0163

[B40] Di Achille P. Tellides G. Humphrey J. D. (2016). Hemodynamics-driven deposition of intraluminal thrombus in abdominal aortic aneurysms. Int. J. Numer. Method. Biomed. Eng. 33:e2828. 10.1002/cnm.2828 27569676 PMC5332472

[B41] Di Martino E. Mantero S. Inzoli F. Melissano G. Astore D. Chiesa R.. (1998). Biomechanics of abdominal aortic aneurysm in the presence of endoluminal thrombus: experimental characterisation and structural static computational analysis. Eur. J. Vasc. Endovasc. Surg. 15, 290–299. 10.1016/S1078-5884(98)80031-2 9610340

[B42] Di Martino E. S. Guadagni G. Fumero A. Ballerini G. Spirito R. Biglioli P.. (2001). Fluid–structure interaction within realistic three-dimensional models of the aneurysmatic aorta as a guidance to assess the risk of rupture of the aneurysm. Med. Eng. Phys. 23, 647–655. 10.1016/S1350-4533(01)00093-5 11755809

[B43] Di Martino E. S. Vorp D. A. (2003). Effect of variation in intraluminal thrombus constitutive properties on abdominal aortic aneurysm wall stress. Ann. Biomed. Eng. 31, 804–809. 10.1114/1.1581880 12971613

[B44] Donea J. Giuliani S. Halleux J. P. (1982). An arbitrary lagrangian-eulerian finite element method for transient dynamic fluid-structure interactions. Comput. Methods Appl. Mech. Eng. 33, 689–723. 10.1016/0045-7825(82)90128-1

[B45] Doyle B. J. Cloonan A. J. Walsh M. T. Vorp D. A. McGloughlin T. M. (2010). Identification of rupture locations in patient-specific abdominal aortic aneurysms using experimental and computational techniques. J. Biomech. 43, 1408–1416. 10.1016/j.jbiomech.2009.09.057 20152982 PMC2857518

[B46] Doyle B. J. Corbett T. J. Callanan A. Walsh M. T. Vorp D. A. McGloughlin T. M. (2009a). An experimental and numerical comparison of the rupture locations of an abdominal aortic aneurysm. J. Endovasc. Ther. 16, 322–335. 10.1583/09-2697.1 19642790 PMC2795364

[B47] Doyle B. J. Corbett T. J. Cloonan A. J. O'Donnell M. R. Walsh M. T. Vorp D. A.. (2009b). Experimental modelling of aortic aneurysms: novel applications of silicone rubbers. Med. Eng. Phys. 31, 1002–1012. 10.1016/j.medengphy.2009.06.002 19595622 PMC2757445

[B48] Doyle B. J. McGloughlin T. M. Miller K. Powell J. T. Norman P. E. (2014). Regions of high wall stress can predict the future location of rupture of abdominal aortic aneurysm. Cardiovasc. Intervent. Radiol. 37, 815–818. 10.1007/s00270-014-0864-7 24554200

[B49] Doyle B. J. Morris L. G. Callanan A. Kelly P. Vorp D. A. McGloughlin T. M. (2008). 3D reconstruction and manufacture of real abdominal aortic aneurysms: from CT scan to silicone model. J. Biomech. Eng. 130:034501. 10.1115/1.2907765 18532870

[B50] Drewe C. J. Parker L. P. Kelsey L. J. Norman P. E. Powell J. T. Doyle B. J. (2017). Haemodynamics and stresses in abdominal aortic aneurysms: a fluid-structure interaction study into the effect of proximal neck and iliac bifurcation angle. J. Biomech. 60, 150–156. 10.1016/j.jbiomech.2017.06.029 28693819

[B51] Duclaux V. Gallaire F. Clanet C. (2010). A fluid mechanical view on abdominal aortic aneurysms. J. Fluid Mech. 664, 5–32. 10.1017/S0022112010003782

[B52] Durst F. Ray S. Ünsal B. Bayoumi O.A. (2005). The development lengths of laminar pipe and channel flows. J. Fluids Eng. 127, 1154–1160. 10.1115/1.2063088

[B53] Egelhoff C. J. Budwig R. S. Elger D. F. Khraishi T. A. Johansen K. H. (1999). Model studies of the flow in abdominal aortic aneurysms during resting and exercise conditions. J. Biomech. 32, 1319–1329. 10.1016/S0021-9290(99)00134-7 10569710

[B54] Ene F. Gachon C. Delassus P. Carroll R. Stefanov F. O'Flynn P.. (2011). *In vitro* evaluation of the effects of intraluminal thrombus on abdominal aortic aneurysm wall dynamics. Med. Eng. Phys. 33, 957–966. 10.1016/j.medengphy.2011.03.005 21478044

[B55] Ene-Iordache B. Remuzzi A. (2012). Disturbed flow in radial-cephalic arteriovenous fistulae for haemodialysis: low and oscillating shear stress locates the sites of stenosis. Nephrol. Dialysis Trans. 27, 358–368. 10.1093/ndt/gfr342 21771751

[B56] Feng Y. Wada S. Ishikawa T. Tsubota K.-I. Yamaguchi T. (2008). A rule-based computational study on the early progression of intracranial aneurysms using fluid-structure interaction: comparison between straight model and curved model. J. Biomech. Sci. Eng. 3, 124–137. 10.1299/jbse.3.124

[B57] Field D. A. (1988). Laplacian smoothing and delaunay triangulations. Commun. Appl. Num. Methods 4, 709–712. 10.1002/cnm.1630040603

[B58] Fillinger M. F. Marra S. P. Raghavan M. L. Kennedy F. E. (2003). Prediction of rupture risk in abdominal aortic aneurysm during observation: wall stress versus diameter. J. Vasc. Surg. 37, 724–732. 10.1067/mva.2003.213 12663969

[B59] Fillinger M. F. Raghavan M. L. Marra S. P. Cronenwett J. L. Kennedy F. E. (2002). *In vivo* analysis of mechanical wall stress and abdominal aortic aneurysm rupture risk. J. Vasc. Surg. 36, 589–597. 10.1067/mva.2002.125478 12218986

[B60] Formaggia L. Lamponi D. Quarteroni A. (2003). One-dimensional models for blood flow in arteries. J. Eng. Math. 47, 251–276. 10.1023/B:ENGI.0000007980.01347.29

[B61] Franck G. Dai J. Fifre A. Ngo S. Justine C. Michineau S.. (2013). Reestablishment of the endothelial lining by endothelial cell therapy stabilizes experimental abdominal aortic aneurysms. Circulation 127, 1877–1887. 10.1161/CIRCULATIONAHA.113.001677 23572502

[B62] Frayne R. Holdsworth D. W. Gowman L. M. Rickey D. W. Drangova M. Fenster A.. (1992). Computer-controlled flow simulator for MR flow studies. J. Magn. Reson. Imaging 2, 605–612. 10.1002/jmri.1880020522 1392256

[B63] Fukushima T. Matsuzawa T. Homma T. (1989). Visualization and finite element analysis of pulsatile flow in models of the abdominal aortic aneurysm. Biorheology 26, 109–130. 10.3233/BIR-1989-26203 2605323

[B64] Gaillard E. Deplano V. (2005). Experimental study of the hemodynamics in an abdominal aortic aneurysm under physiological resting and exercise flow conditions. Comput. Methods Biomech. Biomed. Engin. 8, 109–110. 10.1080/10255840512331388498

[B65] Gao F. Ueda H. Gang L. Okada H. (2013). Fluid structure interaction simulation in three-layered aortic aneurysm model under pulsatile flow: comparison of wrapping and stenting. J. Biomech. 46, 1335–1342. 10.1016/j.jbiomech.2013.02.002 23477789

[B66] Gleason R. L. Humphrey J. D. (2005). Effects of a sustained extension on arterial growth and remodeling: a theoretical study. J. Biomech. 38, 1255–1261. 10.1016/j.jbiomech.2004.06.017 15863110

[B67] Groves E. M. Falahatpisheh A. Su J. L. Kheradvar A. (2014). The effects of positioning of transcatheter aortic valves on fluid dynamics of the aortic root. ASAIO J. 60, 545–552. 10.1097/MAT.0000000000000107 25010918 PMC4334568

[B68] He X. Ku D. N. (1994). Unsteady entrance flow development in a straight tube. J. Biomech. Eng. 116, 355–360. 10.1115/1.2895742 7799639

[B69] Hinnen J.-W. Koning O. H. J. Visser M. J. T. Van Bockel H. J. (2005). Effect of intraluminal thrombus on pressure transmission in the abdominal aortic aneurysm. J. Vasc. Surg. 42, 1176–1182. 10.1016/j.jvs.2005.08.027 16376211

[B70] Ho C. K. Chee A. J. Y. Yiu B. Y. S. Tsang A. C. O. Chow K. W. Yu A. C. H. (2017). Wall-less flow phantoms with tortuous vascular geometries: design principles and a patient-specific model fabrication example. IEEE Trans. Ultrason. Ferroelectr. Freq. Control 64, 25–38. 10.1109/TUFFC.2016.2636129 27959808

[B71] Holzapfel G. A. Gasser T. C. Ogden R. W. (2000). A new constitutive framework for arterial wall mechanics and a comparative study of material models. J. Elasticity Phys. Sci. Solids 61, 1–48. 10.1007/0-306-48389-0_1

[B72] Houard X. Rouzet F. Touat Z. Philippe M. Dominguez M. Fontaine V.. (2007). Topology of the fibrinolytic system within the mural thrombus of human abdominal aortic aneurysms. J. Pathol. 212, 20–28. 10.1002/path.2148 17352452

[B73] Hua J. Mower W. R. (2001). Simple geometric characteristics fail to reliably predict abdominal aortic aneurysm wall stresses. J. Vasc. Surg. 34, 308–315. 10.1067/mva.2001.114815 11496284

[B74] Humphrey J. D. Holzapfel G. A. (2012). Mechanics, mechanobiology, and modeling of human abdominal aorta and aneurysms. J. Biomech. 45, 805–814. 10.1016/j.jbiomech.2011.11.021 22189249 PMC3294195

[B75] Humphrey J. D. Rajagopal K. R. (2003). A constrained mixture model for arterial adaptations to a sustained step change in blood flow. Biomech. Model. Mechanobiol. 2, 109–126. 10.1007/s10237-003-0033-4 14586812

[B76] Humphrey J. D. Taylor C. A. (2008). Intracranial and abdominal aortic aneurysms: similarities, differences, and need for a new class of computational models. Annu. Rev. Biomed. Eng. 10, 221–246. 10.1146/annurev.bioeng.10.061807.160439 18647115 PMC2742216

[B77] Ionita C. N. Mokin M. Varble N. Bednarek D. R. Xiang J. Snyder K. V.. (2014). Challenges and limitations of patient-specific vascular phantom fabrication using 3D Polyjet printing. Proc. SPIE–Int. Soc. Opt. Eng. 9038:90380M. 10.1117/12.2042266 25300886 PMC4188370

[B78] Kandail H. Hamady M. Xu X. Y. (2015). Comparison of blood flow in branched and fenestrated stent-grafts for endovascular repair of abdominal aortic aneurysms. J. Endovasc. Ther. 22, 578–590. 10.1177/1526602815587261 25979146

[B79] Karšaj I. Humphrey J. D. (2012). A multilayered wall model of arterial growth and remodeling. Mech. Mater. 44, 110–119. 10.1016/j.mechmat.2011.05.006 22180692 PMC3237688

[B80] Kelsey L. J. Powell J. T. Norman P. E. Miller K. Doyle B. J. (2016). A comparison of hemodynamic metrics and intraluminal thrombus burden in a common iliac artery aneurysm. Int. J. Numer. Method. Biomed. Eng. 33:e2821. 10.1002/cnm.2821 27509188

[B81] Khanafer K. Berguer R. (2009). Fluid–structure interaction analysis of turbulent pulsatile flow within a layered aortic wall as related to aortic dissection. J. Biomech. 42, 2642–2648. 10.1016/j.jbiomech.2009.08.010 19765711

[B82] Khanafer K. M. Bull J. L. Upchurch G. R. Berguer R. (2007). Turbulence significantly increases pressure and fluid shear stress in an aortic aneurysm model under resting and exercise flow conditions. Ann. Vasc. Surg. 21, 67–74. 10.1016/j.avsg.2006.10.009 17349339

[B83] Khanafer K. M. Gadhoke P. Berguer R. Bull J. L. (2006). Modeling pulsatile flow in aortic aneurysms: effect of non-Newtonian properties of blood. Biorheology 43, 661–679. 17047283

[B84] Kontopodis N. Metaxa E. Papaharilaou Y. Tavlas E. Tsetis D. Ioannou C. (2014). Advancements in identifying biomechanical determinants for abdominal aortic aneurysm rupture. Vascular 23, 65–77. 10.1177/1708538114532084 24757027

[B85] Kose U. Putter S. D. Hoogeveen R. Breeuwer M. (2006). Computational fluid dynamics of abdominal aortic aneurysms with patient-specific inflow boundary conditions, in Medical Imaging: SPIE (San Diego, CA), 11 10.1117/12.649755

[B86] Krishna S. M. Dear A. E. Norman P. E. Golledge J. (2010). Genetic and epigenetic mechanisms and their possible role in abdominal aortic aneurysm. Atherosclerosis 212, 16–29. 10.1016/j.atherosclerosis.2010.02.008 20347091

[B87] Ku D. N. (1997). Blood flow in arteries. Annu. Rev. Fluid Mech. 29, 399–434. 10.1146/annurev.fluid.29.1.399

[B88] Ku D. N. Glagov S. Moore J. E. Zarins C. K. (1989). Flow patterns in the abdominal aorta under simulated postprandial and exercise conditions: an experimental study. J. Vasc. Surg. 9, 309–316. 10.1016/0741-5214(89)90051-7 2918626

[B89] Kung E. O. Les A. S. Medina F. Wicker R. B. McConnell M. V. Taylor C. A. (2011). *In vitro* validation of finite-element model of AAA hemodynamics incorporating realistic outlet boundary conditions. J. Biomech. Eng. 133:041003. 10.1115/1.4003526 21428677 PMC4404703

[B90] Lasheras J. C. (2006). The Biomechanics of Arterial Aneurysms. Annu. Rev. Fluid Mech. 39, 293–319. 10.1146/annurev.fluid.39.050905.110128

[B91] Les A. S. Shadden S. C. Figueroa C. A. Park J. M. Tedesco M. M. Herfkens R. J.. (2010). Quantification of hemodynamics in abdominal aortic aneurysms during rest and exercise using magnetic resonance imaging and computational fluid dynamics. Ann. Biomed. Eng. 38, 1288–1313. 10.1007/s10439-010-9949-x 20143263 PMC6203348

[B92] Longo C. Upchurch G. R. (2005). Abdominal aortic aneurysm screening: recommendations and controversies. Vasc. Endovascular Surg. 39, 213–219. 10.1177/153857440503900301 15920649

[B93] Mandal P. K. (2005). An unsteady analysis of non-Newtonian blood flow through tapered arteries with a stenosis. Int. J. Non Linear Mech. 40, 151–164. 10.1016/j.ijnonlinmec.2004.07.007

[B94] McGloughlin Timothy M. Doyle Barry J. (2010). New approaches to abdominal aortic aneurysm rupture risk assessment. Arterioscler. Thromb. Vasc. Biol. 30, 1687–1694. 10.1161/ATVBAHA.110.204529 20508202

[B95] Mechoor R. R. Schmidt T. Kung E. (2016). A real-time programmable pulsatile flow pump for *in vitro* cardiovascular experimentation. J. Biomech. Eng. 138:111002. 10.1115/1.4034561 27590025

[B96] Meyer C. A. Bertrand E. Boiron O. Deplano V. (2011). Stereoscopically observed deformations of a compliant abdominal aortic aneurysm model. J. Biomech. Eng. 133:111004. 10.1115/1.4005416 22168736

[B97] Mohamied Y. Rowland E. M. Bailey E. L. Sherwin S. J. Schwartz M. A. Weinberg P. D. (2015). Change of direction in the biomechanics of atherosclerosis. Ann. Biomed. Eng. 43, 16–25. 10.1007/s10439-014-1095-4 25138165 PMC4286626

[B98] Moore J. J. E. Ku D. N. (1994). Pulsatile velocity measurements in a model of the human abdominal aorta under simulated exercise and postprandial conditions. J. Biomech. Eng. 116, 107–111. 10.1115/1.2895692 8189705

[B99] Moore J. J. E. Ku D. N. Zarins C. K. Glagov S. (1992). Pulsatile flow visualization in the abdominal aorta under differing physiologic conditions: implications for increased susceptibility to atherosclerosis. J. Biomech. Eng. 114, 391–397. 10.1115/1.2891400 1295493

[B100] Morris L. Stefanov F. McGloughlin T. (2013). Stent graft performance in the treatment of abdominal aortic aneurysms: The influence of compliance and geometry. J. Biomech. 46, 383–395. 10.1016/j.jbiomech.2012.11.026 23218139

[B101] Mower W. R. Quiñones W. J. Gambhir S. S. (1997). Effect of intraluminal thrombus on abdominal aortic aneurysm wall stress. J. Vasc. Surg. 26, 602–608. 10.1016/S0741-5214(97)70058-2 9357460

[B102] Najjari M. R. Plesniak M. W. (2016). Evolution of vortical structures in a curved artery model with non-Newtonian blood-analog fluid under pulsatile inflow conditions. Exp. Fluids 57:100 10.1007/s00348-016-2188-7

[B103] Nerem R. M. (1984). Atherogenesis: hemodynamics, vascular geometry, and the endothelium. Biorheology 21, 565–569. 10.3233/BIR-1984-21415 6487767

[B104] Nikolaidis N. M. Mathioulakis D. S. (2002). Axial and secondary flow study in a 90 deg bifurcation under pulsating conditions using PIV. J. Fluids Eng. 124, 505–511. 10.1115/1.1470478

[B105] O'Leary S. Kavanagh E. Grace P. McGloughlin T. Doyle B. (2013). Determination of layer and region specific mechanical properties of intraluminal thrombus (ILT): the importance of biaxial tensile testing, in ASME 2013 Summer Bioengineering Conference (Sunriver, OR). 10.1115/SBC2013-14237

[B106] O'Leary S. A. Kavanagh E. G. Grace P. A. McGloughlin T. M. Doyle B. J. (2014). The biaxial mechanical behaviour of abdominal aortic aneurysm intraluminal thrombus: Classification of morphology and the determination of layer and region specific properties. J. Biomech. 47, 1430–1437. 10.1016/j.jbiomech.2014.01.041 24565182

[B107] O'Leary S. A. Mulvihill J. J. Barrett H. E. Kavanagh E. G. Walsh M. T. McGloughlin T. M.. (2015). Determining the influence of calcification on the failure properties of abdominal aortic aneurysm (AAA) tissue. J. Mech. Behav. Biomed. Mater. 42, 154–167. 10.1016/j.jmbbm.2014.11.005 25482218

[B108] O'Rourke M. J. McCullough J. P. (2010). An investigation of the flow field within patient-specific models of an abdominal aortic aneurysm under steady inflow conditions. Proc.Inst. Mech. Eng. H 224, 971–988. 10.1243/09544119JEIM694 20923115

[B109] O'Rourke M. J. McCullough J. P. Kelly S. (2012). An investigation of the relationship between hemodynamics and thrombus deposition within patient-specific models of abdominal aortic aneurysm. Proc. Inst. Mech. Eng. Part H 226, 548–564. 10.1177/0954411912444080 22913102

[B110] Owen B. Lowe C. Ashton N. Mandal P. Rogers S. Wein W.. (2016). Computational hemodynamics of abdominal aortic aneurysms: three-dimensional ultrasound versus computed tomography. Proc. Inst. Mech. Eng. Part H 230, 201–210. 10.1177/0954411915626742 26893226

[B111] Pahlevan N. M. Gharib M. (2013). *In-vitro* investigation of a potential wave pumping effect in human aorta. J. Biomech. 46, 2122–2129. 10.1016/j.jbiomech.2013.07.006 23915578

[B112] Papaharilaou Y. Ekaterinaris J. A. Manousaki E. Katsamouris A. N. (2007). A decoupled fluid structure approach for estimating wall stress in abdominal aortic aneurysms. J. Biomech. 40, 367–377. 10.1016/j.jbiomech.2005.12.013 16500664

[B113] Peattie R. A. Asbury C. L. Bluth E. I. Riehle T. J. (1996). Steady flow in models of abdominal aortic aneurysms. Part II: Wall stresses and their implication for *in vivo* thrombosis and rupture. J. Ultrasound Med. 15, 689–696. 10.7863/jum.1996.15.10.689 8887240

[B114] Peattie R. A. Riehle T. J. Bluth E. I. (2004). Pulsatile flow in fusiform models of abdominal aortic aneurysms: flow fields, velocity patterns and flow-induced wall stresses. J. Biomech. Eng. 126, 438–446. 10.1115/1.1784478 15543861

[B115] Poelma C. Watton Paul N. Ventikos Y. (2015). Transitional flow in aneurysms and the computation of haemodynamic parameters. J. R. Soc. Interface 12:20141394. 10.1098/rsif.2014.1394 25694540 PMC4387528

[B116] Poepping T. L. Nikolov H. N. Thorne M. L. Holdsworth D. W. (2004). A thin-walled carotid vessel phantom for Doppler ultrasound flow studies. Ultrasound Med. Biol. 30, 1067–1078. 10.1016/j.ultrasmedbio.2004.06.003 15474751

[B117] Qiu Y. Yuan D. Wen J. Fan Y. Zheng T. (2018). Numerical identification of the rupture locations in patient-specific abdominal aortic aneurysmsusing hemodynamic parameters. Comput. Methods Biomech. Biomed. Engin. 21, 1–12. 10.1080/10255842.2017.1410796 29251991

[B118] Raghavan M. Kratzberg J. da Silva E. S.o. (2004). Heterogeneous, variable wall-thickness modeling of a ruptured abdominal aortic aneurysm, in ASME International Mechanical Engineering Congress and Exposition (Anaheim, CA), 271–272. 10.1115/IMECE2004-60018

[B119] Raghavan M. L. Kratzberg J. Castro de Tolosa E. M. Hanaoka M. M. Walker P. da Silva E. S. (2006). Regional distribution of wall thickness and failure properties of human abdominal aortic aneurysm. J. Biomech. 39, 3010–3016. 10.1016/j.jbiomech.2005.10.021 16337949

[B120] Raghavan M. L. Vorp D. A. (2000). Toward a biomechanical tool to evaluate rupture potential of abdominal aortic aneurysm: identification of a finite strain constitutive model and evaluation of its applicability. J. Biomech. 33, 475–482. 10.1016/S0021-9290(99)00201-8 10768396

[B121] Raghavan M. L. Vorp D. A. Federle M. P. Makaroun M. S. Webster M. W. (2000). Wall stress distribution on three-dimensionally reconstructed models of human abdominal aortic aneurysm. J. Vasc. Surg. 31, 760–769. 10.1067/mva.2000.103971 10753284

[B122] Raptis A. Xenos M. Georgakarakos E. Kouvelos G. Giannoukas A. Matsagkas M. (2017). Hemodynamic profile of two aortic endografts accounting for their postimplantation position. J. Med. Devices 11:021003 10.1115/1.4035687

[B123] Rivlin R. S. Saunders D. (1951). Large elastic deformations of isotropic materials VII. Experiments on the deformation of rubber. Philos. Trans. R. Soc. Lond. Ser. A Math. Phys. Sci. 243, 251–288. 10.1098/rsta.1951.0004

[B124] Roloff C. Berg P. Redel T. Janiga G. Thévenin D. (2017). Tomographic particle image velocimetry for the validation of hemodynamic simulations in an intracranial aneurysm, in 2017 39th Annual International Conference of the IEEE Engineering in Medicine and Biology Society (EMBC) (Jeju Island), 1340–1343. 10.1109/EMBC.2017.8037080 29060124

[B125] Roloff C. Stucht D. Beuing O. Berg P. (2018). Comparison of intracranial aneurysm flow quantification techniques: standard PIV vs. stereoscopic PIV vs. tomographic PIV vs. phase-contrast MRI vs. CFD. J. Neurointerven. Surg. Neurintsurg. 11, 275–282. 10.1136/neurintsurg-2018-013921 30061369

[B126] Sakalihasan N. Limet R. Defawe O. D. (2005). Abdominal aortic aneurysm. Lancet 365, 1577–1589. 10.1016/S0140-6736(05)66459-8 15866312

[B127] Salsac A.-V. Sparks S. R. Chomaz J.-M. Lasheras J. C. (2006). Evolution of the wall shear stresses during the progressive enlargement of symmetric abdominal aortic aneurysms. J. Fluid Mech. 560, 19–51. 10.1017/S002211200600036X

[B128] Scherer P. W. (1973). Flow in axisymmetrical glass model aneurysms. J. Biomech. 6, 695–700. 10.1016/0021-9290(73)90025-0 4757487

[B129] Scott R. A. P. Ashton H. A. Lamparelli M. J. Harris G. J. C. Stevens J. W. (2002). A 14-year experience with 6 cm as a criterion for surgical treatment of abdominal aortic aneurysm. BJS 86, 1317–1321. 10.1046/j.1365-2168.1999.01227.x 10540141

[B130] Scotti C. M. Finol E. A. (2007). Compliant biomechanics of abdominal aortic aneurysms: a fluid–structure interaction study. Comput. Struct. 85, 1097–1113. 10.1016/j.compstruc.2006.08.041

[B131] Scotti C. M. Jimenez J. Muluk S. C. Finol E. A. (2008). Wall stress and flow dynamics in abdominal aortic aneurysms: finite element analysis vs. fluid–structure interaction. Comput. Methods Biomech. Biomed. Eng. 11, 301–322. 10.1080/10255840701827412 18568827

[B132] Scotti C. M. Shkolnik A. D. Muluk S. C. Finol E. A. (2005). Fluid-structure interaction in abdominal aortic aneurysms: effects of asymmetry and wall thickness. Biomed. Eng. Online 4:64. 10.1186/1475-925X-4-64 16271141 PMC1298313

[B133] Shadden S. C. Arzani A. (2015). Lagrangian postprocessing of computational hemodynamics. Ann. Biomed. Eng. 43, 41–58. 10.1007/s10439-014-1070-0 25059889 PMC4289096

[B134] Simão da Silva E. Rodrigues A. J. Magalhães Castro de Tolosa E. Rodrigues C. J. Villas Boas do Prado G. Nakamoto J. C. (2000). Morphology and diameter of infrarenal aortic aneurysms: a prospective autopsy study. Cardiovasc. Surg. 8, 526–532. 10.1016/S0967-2109(00)00060-0 11068212

[B135] Simsek F. G. Kwon Y. W. (2015). Investigation of material modeling in fluid–structure interaction analysis of an idealized three-layered abdominal aorta: aneurysm initiation and fully developed aneurysms. J. Biol. Phys. 41, 173–201. 10.1007/s10867-014-9372-x 25624113 PMC4366435

[B136] Singh J. Brunner G. Morrisett J. D. Ballantyne C. M. Lumsden A. B. Shah D. J. (2018). Patient-specific flow descriptors and normalised wall index in peripheral artery disease: a preliminary study. Comput. Methods Biomech. Biomed. Eng. 6, 119–127. 10.1080/21681163.2016.1184589 PMC5830147 29503774

[B137] Soudah E. Ng E. Y. K. Loong T. H. Bordone M. Pua U. Narayanan S. (2013). CFD modelling of abdominal aortic aneurysm on hemodynamic loads using a realistic geometry with CT. Comput. Math. Methods Med. 2013, 472564–472564. 10.1155/2013/472564 23864906 PMC3707263

[B138] Stamatopoulos C. Mathioulakis D. S. Papaharilaou Y. Katsamouris A. (2011). Experimental unsteady flow study in a patient-specific abdominal aortic aneurysm model. Exp. Fluids 50, 1695–1709. 10.1007/s00348-010-1034-6

[B139] Stamatopoulos C. Papaharilaou Y. Mathioulakis D. S. Katsamouris A. (2010). Steady and unsteady flow within an axisymmetric tube dilatation. Exp. Thermal Fluid Sci. 34, 915–927. 10.1016/j.expthermflusci.2010.02.008

[B140] Stamhuis E. J. (2006). Basics and principles of particle image velocimetry (PIV) for mapping biogenic and biologically relevant flows. Aquatic Ecol. 40, 463–479. 10.1007/s10452-005-6567-z

[B141] Stringfellow M. M. Lawrence P. F. Stringfellow R. G. (1987). The influence of aorta-aneurysm geometry upon stress in the aneurysm wall. J. Surg. Res. 42, 425–433. 10.1016/0022-4804(87)90178-8 3573768

[B142] Sughimoto K. Takahara Y. Mogi K. Yamazaki K. Tsubota K. I. Liang F.. (2014). Blood flow dynamic improvement with aneurysm repair detected by a patient-specific model of multiple aortic aneurysms. Heart Vessels 29, 404–412. 10.1007/s00380-013-0381-7 23852404

[B143] Sulaiman A. Roty C. Serfaty J. M. Attia C. Huet L. Douek P. (2008). *In vitro*, nonrigid model of aortic arch aneurysm. J. Vasc. Interven. Radiol. 19, 919–924. 10.1016/j.jvir.2008.02.009 18503908

[B144] Sun N. Leung J. H. Wood N. B. Hughes A. D. Thom S. A. Cheshire N. J.. (2009). Computational analysis of oxygen transport in a patient-specific model of abdominal aortic aneurysm with intraluminal thrombus. Br. J. Radiol. 82, S18–S23. 10.1259/bjr/89466318 20348531

[B145] Swedenborg J. Eriksson P. E. R. (2006). The intraluminal thrombus as a source of proteolytic activity. Ann. N. Y. Acad. Sci. 1085, 133–138. 10.1196/annals.1383.044 17182929

[B146] Tang D. Yang C. Zheng J. Woodard P. K. Saffitz J. E. Sicard G. A.. (2005). Quantifying effects of plaque structure and material properties on stress distributions in human atherosclerotic plaques using 3D FSI models. J. Biomech. Eng. 127, 1185–1194. 10.1115/1.2073668 16502661 PMC1474006

[B147] Tanweer O. Wilson T. A. Metaxa E. Riina H. A. Meng H. (2014). A comparative review of the hemodynamics and pathogenesis of cerebral and abdominal aortic aneurysms: lessons to learn from each other. J. Cerebrovasc. Endovasc. Neurosurg. 16, 335–349. 10.7461/jcen.2014.16.4.335 25599042 PMC4296046

[B148] Taubin G. (1995). Curve and surface smoothing without shrinkage, in Proceedings of IEEE International Conference on Computer Vision (Cambridge, MA), 852–857. 10.1109/ICCV.1995.466848

[B149] Thurston G. B. (1979). Rheological parameters for the viscosity viscoelasticity and thixotropy of blood. Biorheology 16, 149–162. 10.3233/BIR-1979-16303 508925

[B150] Tong J. Holzapfel G. A. (2015). Structure, mechanics, and histology of intraluminal thrombi in abdominal aortic aneurysms. Ann. Biomed. Eng. 43, 1488–1501. 10.1007/s10439-015-1332-5 25986953

[B151] Tsai W. Savaş Ö. (2010). Flow pumping system for physiological waveforms. Med. Biol. Eng. Comput. 48, 197–201. 10.1007/s11517-009-0573-6 20052555 PMC2807595

[B152] Valentín A. Humphrey J. D. Holzapfel G. A. (2011). A multi-layered computational model of coupled elastin degradation, vasoactive dysfunction, and collagenous stiffening in aortic aging. Ann. Biomed. Eng. 39, 2027–2045. 10.1007/s10439-011-0287-4 21380570 PMC3251773

[B153] Valentín A. Humphrey J. D. Holzapfel G. A. (2013). A finite element-based constrained mixture implementation for arterial growth, remodeling, and adaptation: Theory and numerical verification. Int. J. Numer. Method. Biomed. Eng. 29, 822–849. 10.1002/cnm.2555 23713058 PMC3735847

[B154] van ‘t Veer M. Buth J. Merkx M. Tonino P. van den Bosch H. Pijls N.. (2008). Biomechanical properties of abdominal aortic aneurysms assessed by simultaneously measured pressure and volume changes in humans. J. Vasc. Surg. 48, 1401–1407. 10.1016/j.jvs.2008.06.060 18771885

[B155] Vande Geest J. P. Sacks M. S. Vorp D. A. (2006a). The effects of aneurysm on the biaxial mechanical behavior of human abdominal aorta. J. Biomech. 39, 1324–1334. 10.1016/j.jbiomech.2005.03.003 15885699

[B156] Vande Geest J. P. Sacks M. S. Vorp D. A. (2006b). A planar biaxial constitutive relation for the luminal layer of intra-luminal thrombus in abdominal aortic aneurysms. J. Biomech. 39, 2347–2354. 10.1016/j.jbiomech.2006.05.011 16872617

[B157] Vollmer J. Mencl R. Müller H. (2001). Improved laplacian smoothing of noisy surface meshes. Computer Graphics Forum 18, 131–138. 10.1111/1467-8659.00334

[B158] Vorp D. A. Raghavan M. L. Muluk S. C. Makaroun M. S. Steed D. L. Shapiro R. O. N.. (1996). Wall strength and stiffness of aneurysmal and nonaneurysmal abdominal aorta. Ann. N. Y. Acad. Sci. 800, 274–276. 10.1111/j.1749-6632.1996.tb33330.x 8959012

[B159] Vorp D. A. Raghavan M. L. Webster M. W. (1998). Mechanical wall stress in abdominal aortic aneurysm: influence of diameter and asymmetry. J. Vasc. Surg. 27, 632–639. 10.1016/S0741-5214(98)70227-7 9576075

[B160] Wang Y. Joannic D. Patrick J. Keromnes A. Aurélien M. Lalande A. (2016). Comparison of Flow Measurement by 4D Flow Magnetic Resonance Imaging and by Particles Image Velocimetry on Phantom of Abdominal Aortic Aneurysm. SM Vasc Med, SM Online Scientific Resources LLC.

[B161] Watton P. N. Hill N. A. Heil M. (2004). A mathematical model for the growth of the abdominal aortic aneurysm. Biomech. Model. Mechanobiol. 3, 98–113. 10.1007/s10237-004-0052-9 15452732

[B162] Watts D. M. Sutcliffe C. J. Morgan R. H. Meagher S. Wardlaw J. Connell M.. (2007). Anatomical flow phantoms of the nonplanar carotid bifurcation, Part I: computer-aided design and fabrication. Ultrasound Med. Biol. 33, 296–302. 10.1016/j.ultrasmedbio.2006.08.003 17306699

[B163] Wilson J. S. Baek S. Humphrey J. D. (2013). Parametric study of effects of collagen turnover on the natural history of abdominal aortic aneurysms. Proc. R. Soc. A 469:20120556. 10.1098/rspa.2012.0556 23633905 PMC3637002

[B164] Wolters B. J. B. M. Rutten M. C. M. Schurink G. W. H. Kose U. de Hart J. van de Vosse F. N. (2005). A patient-specific computational model of fluid–structure interaction in abdominal aortic aneurysms. Med. Eng. Phys. 27, 871–883. 10.1016/j.medengphy.2005.06.008 16157501

[B165] Womersley J. R. (1955). Method for the calculation of velocity, rate of flow and viscous drag in arteries when the pressure gradient is known. J. Physiol. 127, 553–563. 10.1113/jphysiol.1955.sp005276 14368548 PMC1365740

[B166] Wong E. Y. Thorne M. L. Nikolov H. N. Poepping T. L. Holdsworth D. W. (2008). Doppler ultrasound compatible plastic material for use in rigid flow models. Ultrasound Med. Biol. 34, 1846–1856. 10.1016/j.ultrasmedbio.2008.01.002 18343018

[B167] Wu J. Shadden S. C. (2015). Coupled simulation of hemodynamics and vascular growth and remodeling in a subject-specific geometry. Ann. Biomed. Eng. 43, 1543–1554. 10.1007/s10439-015-1287-6 25731141 PMC4497867

[B168] Xenos M. Alemu Y. Zamfir D. Einav S. Ricotta J. J. Labropoulos N.. (2010). The effect of angulation in abdominal aortic aneurysms: fluid–structure interaction simulations of idealized geometries. Med. Biol. Eng. Comput. 48, 1175–1190. 10.1007/s11517-010-0714-y 21088917

[B169] Yip T. H. Yu S. C. M. (2001). Cyclic transition to turbulence in rigid abdominal aortic aneurysm models. Fluid Dyn. Res. 29:81 10.1016/S0169-5983(01)00018-1

[B170] Yip T. H. Yu S. C. M. (2002). Oscillatory flows in straight tubes with an axisymmetric bulge. Exp. Thermal Fluid Sci. 26, 947–961. 10.1016/S0894-1777(02)00214-5

[B171] Young D. F. (1979). Fluid mechanics of arterial stenoses. J. Biomech. Eng. 101, 157–175. 10.1115/1.3426241

[B172] Yu S. C. M. (2000). Steady and pulsatile flow studies in abdominal aortic aneurysm models using particle image velocimetry. Int. J. Heat Fluid Flow 21, 74–83. 10.1016/S0142-727X(99)00058-2

[B173] Yu S. C. M. Zhao J. B. (2000). A particle image velocimetry study on the pulsatile flow characteristics in straight tubes with an asymmetric bulge. Proc. Inst. Mech. Eng. Part C 214, 655–671. 10.1243/0954406001523678

[B174] Zhang H. Zhang X. Ji S. Guo Y. Ledezma G. Elabbasi N. (2003). Recent development of fluid–structure interaction capabilities in the ADINA system. Comput. Struct. 81, 1071–1085. 10.1016/S0045-7949(03)00009-9

[B175] Zhou J. Adrian R. J. Balachandar S. Kendall T. M. (1999). Mechanisms for generating coherent packets of hairpin vortices in channel flow. J. Fluid Mech. 387, 353–396. 10.1017/S002211209900467X

